# Aspartyl Protease 5 Matures Dense Granule Proteins That Reside at the Host-Parasite Interface in Toxoplasma gondii

**DOI:** 10.1128/mBio.01796-18

**Published:** 2018-10-30

**Authors:** Michael J. Coffey, Laura F. Dagley, Simona Seizova, Eugene A. Kapp, Giuseppe Infusini, David S. Roos, Justin A. Boddey, Andrew I. Webb, Christopher J. Tonkin

**Affiliations:** a The Walter and Eliza Hall Institute of Medical Research, Melbourne, Australia; b Department of Medical Biology, The University of Melbourne, Melbourne, Australia; c Department of Biology, University of Pennsylvania, Philadelphia, Pennsylvania, USA; Washington University School of Medicine

**Keywords:** effector, proteomics, *Toxoplasma*, virulence

## Abstract

Toxoplasma gondii infects approximately 30% of the world’s population, causing disease primarily during pregnancy and in individuals with weakened immune systems. *Toxoplasma* secretes and exports effector proteins that modulate the host during infection, and several of these proteins are processed by the Golgi-associated aspartyl protease 5 (ASP5). Here, we identify ASP5 substrates by selectively enriching N-terminally derived peptides from wild-type and *Δasp5* parasites. We reveal more than 2,000 unique *Toxoplasma* N-terminal peptides, mapping to both natural N termini and protease cleavage sites. Several of these peptides mapped directly downstream of the characterized ASP5 cleavage site, arginine-arginine-leucine (RRL). We validate candidates as true ASP5 substrates, revealing they are not processed in parasites lacking ASP5 or in wild-type parasites following mutation of the motif from RRL to ARL. All identified ASP5 substrates are dense granule proteins, and interestingly, none appear to be exported, thus differing from the analogous system in related *Plasmodium* spp. Instead we show that the majority of substrates reside within the parasitophorous vacuole (PV), and its membrane (the PVM), including two kinases and one phosphatase. We show that genetic deletion of WNG2 leads to attenuation in a mouse model, suggesting that this putative kinase is a new virulence factor in *Toxoplasma*. Collectively, these data constitute the first in-depth analyses of ASP5 substrates and shed new light on the role of ASP5 as a maturase of dense granule proteins during the *Toxoplasma* lytic cycle.

## Abstract (precis)

Toxoplasma gondii is one of the most successful human parasites. Central to its success is the arsenal of virulence proteins introduced into the infected host cell. Several of these virulence proteins require direct maturation by the aspartyl protease ASP5, and all require ASP5 for translocation into the host cell, yet the true number of ASP5 substrates and complete repertoire of effectors is currently unknown. Here we selectively enrich N-terminally derived peptides using Terminal Amine Isotopic Labeling of Substrates (TAILS) and use quantitative proteomics to reveal novel ASP5 substrates. We identify, using two different enrichment techniques, new ASP5 substrates and their specific cleavage sites. ASP5 substrates include two kinases and one phosphatase that reside at the host-parasite interface, which are important for infection.

## INTRODUCTION

Apicomplexan parasites are the causative agents of many diseases of important medical and agricultural significance. As obligate intracellular pathogens, these parasites must invade and then survive within the infected host cell while obtaining nutrients in order to replicate. Toxoplasma gondii is the most widespread and successful of all apicomplexan parasites and resides in nucleated cells of nearly all warm-blooded organisms, including birds and mammals. Initial infection in immunocompetent humans is generally mild; however, some highly virulent South American strains of *Toxoplasma* exist and cause progressive blindness in otherwise healthy individuals (1, 2). Further, reactivation of latent infection within immunocompromised individuals, such as AIDS and immunotherapy patients, can cause severe disease and death (3).

Central to the success of *Toxoplasma* is its ability to modulate the host response, a process achieved through the secretion and export of effector proteins. Subversion of the host enables the parasites to survive and proliferate within the parasitophorous vacuole (PV), a structure that delimits *Toxoplasma* from the hostile intracellular environment. At the molecular level, *Toxoplasma* achieves this fine balance of immune modulation by the orchestrated secretion of effector proteins using two separate protein trafficking pathways. Proteins from the rhoptry organelles (termed ROPs) are introduced during the invasion process where they simultaneously promote a proinflammatory microenvironment to protect the host from excessive parasite growth, while also ensuring *Toxoplasma* is not cleared by the mounting immune response (4–12).

More recently, it has been shown that another protein export pathway exists. Here, dense granule proteins (GRAs) are secreted postinvasion and can modulate the host either by localizing to the PV membrane (PVM) or by translocating across this barrier and residing within the host cell. GRA15, for example, localizes to the host cytosolic side of the PVM where it activates the host NFκB pathway to induce a protective immune response against the parasite (13), while the transmembrane protein MAF1 recruits host mitochondria to the PVM (14).

Another group of dense granule proteins are translocated across the PVM and into the host cell. GRA16 traffics to the host cell nucleus where it interacts with the phosphatase PP2A (protein phosphatase 2A) and the ubiquitin protease HAUSP, potentially interfering with the cell cycle to avoid premature immune detection (15, 16). GRA24, on the other hand, induces a protective response through the prolonged activation of the mitogen-activated protein kinase (MAPK) protein p38α (16, 17). One of the major subversion mechanisms of *Toxoplasma* is the loss of the infected host cell’s ability to mount a gamma interferon (IFN-γ) response. This response is circumvented by the dense granule protein IST (inhibitor of STAT1-dependent transcription). IST directly binds to activated STAT1 in the host nucleus and recruits a chromatin remodelling complex to block the transcription of STAT1-dependent promoters (18, 19). GRA28 was discovered in a screen by tagging a dense granule protein with the promiscuous biotin ligase BirA, although its role in the host nucleus has not yet been elucidated (20).

The precise mechanism of how *Toxoplasma* proteins translocate across the PVM is currently unclear; however, the export of GRA16, GRA24, and IST is dependent on the Golgi-resident aspartyl protease ASP5, which is known to directly process the exported protein GRA16, and likely IST. Despite not proteolytically maturing GRA24 (18, 21–23), export of this effector still requires the activity of ASP5. ASP5 cleaves its substrates at a defined motif termed the TEXEL (*Toxoplasma* Export Element), a conserved motif consisting of RRLxx, so named as it is homologous to the cleavage of the *Plasmodium* export element (PEXEL) (commonly RxLxE/Q/D) by the endoplasmic reticulum (ER)-resident protease plasmepsin V (PMV) (21, 24–28). Within malaria parasites, the PEXEL appears to occur only near the N terminus of the protein, and its cleavage by PMV somehow licenses proteins for export across the PVM via the *Plasmodium* translocon of exported proteins (PTEX) (25, 29–37).

Despite similarities, there are also several key differences between these pathways in *Toxoplasma* and *Plasmodium*. ASP5 resides within the Golgi, where it cleaves the TEXEL that can be found at the N or C terminus of the substrate. Further, while all reported PEXEL proteins are exported, it appears only a subset of ASP5 substrates are translocated into the host cell. We recently reported that ASP5 processes the PVM protein MYR1, a likely component of the *Toxoplasma* translocon (21, 38). Further, previous work by us and others demonstrated that ASP5-dependent effectors substantially alter the transcriptional profile of the host cell (39). Due to its central role in host cell subversion, parasites lacking ASP5 exhibit decreased virulence in mice, even in the normally lethal RH strain (21, 23).

Despite the importance of ASP5, only a handful of ASP5 substrates have been identified, and the true number of substrates remains unknown. Indeed, it is likely that there are still many unidentified effectors. Identification of new ASP5 substrates could identify new effector proteins and virulence factors driving the persistence of *Toxoplasma*. To identify new ASP5-dependent effector proteins, we have utilized a quantitative proteomic pipeline in combination with the selective enrichment of N-terminally derived peptides. These methods included Terminal Amine Isotopic Labeling of Substrates (TAILS) (40) and Hydrophobic Tagging-Assisted N-Terminal Enrichment (HYTANE) (41), which enabled us to compare differences in the N-terminome between wild-type (WT) and *Δasp5* tachyzoites. Enrichment of N-terminal peptides by TAILS and HYTANE has enabled the identification of protease cleavage sites dependent on ASP5. Moreover, we validated the N-terminal enrichment data and report that ASP5 matures several new dense granule proteins that appear to be localized within the confines of the parasitophorous vacuole. We present LCAT (42) and a new dense granule protein, GRA45, as ASP5 substrates that are processed close to the C-terminal end of the polypeptide. Further, we validate WNG1 and WNG2, formerly ROP35 and ROP34, as two new dense granule protein kinases processed by ASP5. This study greatly increases the number of known ASP5 substrates and demonstrates the role that this protease plays during *Toxoplasma* pathogenesis.

## RESULTS

### Identification of ASP5 substrates by N-terminal peptide enrichment.

**FIG 1:**
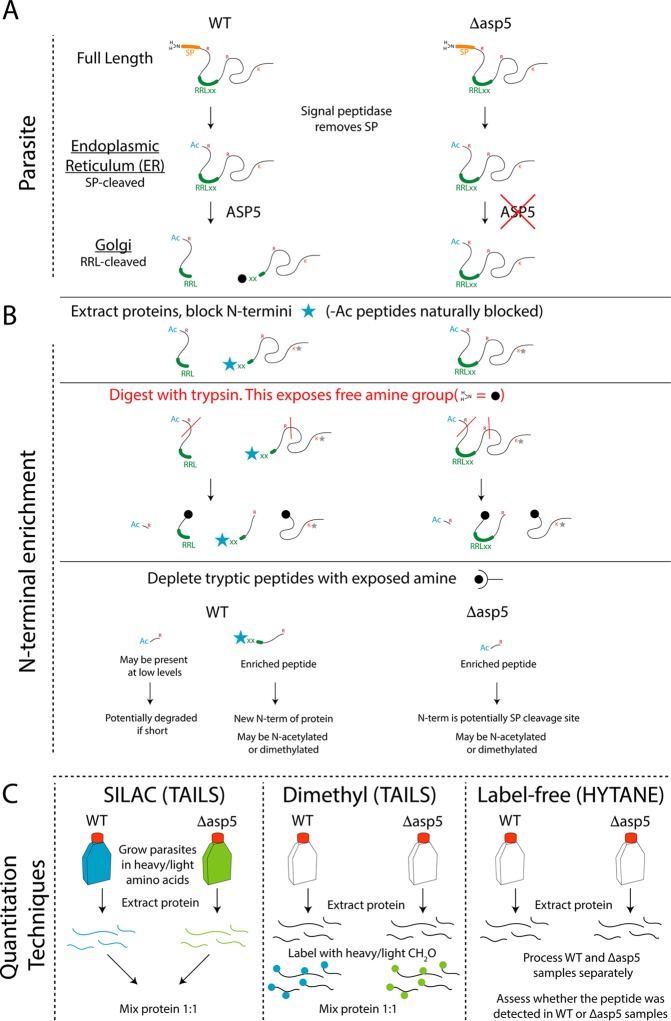
Outline of experimental techniques for identification of N-terminal peptides. (A) Schematic of TEXEL protein processing within WT (left) and *Δasp5* (right) parasites. (B) Following protein extraction, exposed N termini (i.e., containing a free amino group, -NH_2_ [black sphere]) were blocked following the addition of formaldehyde with a catalyst, resulting in primary amine dimethylation. Dimethylation of the most N-terminal amine is depicted by a blue star [-N(CH_3_)_2_], while those on the R chain of lysine residues are depicted by a gray star. Naturally blocked N termini, such as acetylated amines, did not undergo this reaction. The blocked N-terminal peptides were then liberated from their respective proteins by digestion with trypsin. Resulting tryptic peptides (containing an exposed NH_2_ [black sphere]) were then depleted from the mixture either using the synthetic HPG-ALD polymer (TAILS) or using hexadecanal (HYTANE), while the blocked peptides remained in solution; these were then desalted and run on the mass spectrometer. (C) Quantitation techniques. SILAC samples (*n* = 2 reciprocally labeled samples) were mixed prior to beginning the TAILS protocol, while dimethyl samples (*n* = 2 reciprocally labeled samples) were mixed following heavy or light dimethylation of primary amines on day 2 of this protocol. Label-free samples were processed separately (*n* = 9 replicates per group), and neo-N termini were depleted with the HYTANE protocol. WT, wild type; Δ*asp5*, parasites lacking the enzyme ASP5; SP, signal peptide; NH_2_, amine group; RRLxx, TEXEL motif cleaved by ASP5; ASP5, aspartyl protease 5; Ac, acetylated N terminus; R, arginine; K, lysine; blue star, dimethylated [N(CH_3_)_2_] N-terminal amino acid; red line, tryptic cleavage site (after R); black sphere, exposed NH_2_ group on the N terminus; gray star, dimethylated R chain of lysine.

In our current model, we propose that *Toxoplasma* effectors enter the ER via an N-terminal signal peptide that is subsequently cleaved by signal peptidase. Proteolytic cleavage can result in acetylation (or not) of the new N termini (Fig. 1A). Upon reaching the Golgi apparatus, ASP5 matures substrates at the TEXEL (RRLxx), prior to transport across the parasite plasma membrane (PPM) (Fig. 1A). Within the PV space, some substrates are inserted into the PVM, while others are exported into the host cell. Parasites that lack ASP5 are unable to mature these substrates within the Golgi, resulting in different N termini, commonly represented as the signal peptide cleavage site (Fig. 1A).

To discover new ASP5 substrates, we used three unbiased quantitative proteomics approaches (SILAC, heavy dimethyl labeling, and label-free quantitation) in combination with two techniques to enrich N-terminally derived peptides (TAILS and HYTANE) (Fig. 1B). Both techniques rely on formaldehyde-based dimethylation to block free amines on the N termini of proteins and side chains of lysine residues. Second, proteins are enzymatically digested with trypsin, liberating internal tryptic peptides (which have “free” nonacetylated N termini) from N-terminal peptides with blocked amines. The internal peptides are then depleted using an aldehyde-derived polymer (TAILS) or hexadecanal, an amine-reactive reagent (HYTANE), thus enriching for N-terminal peptides. Subsequent mass spectrometry analyses of these N-terminal peptides allow for quantitative differences to be measured in an unbiased fashion, thus enabling the identification of protease cleavage sites when used in combination with protease-deficient genetic mutants.

### Quantitative proteomic approaches to identify ASP5 substrates.

We used SILAC, differential dimethylation, and label-free methodologies to quantitate N-terminally derived peptide abundance between wild-type and ASP5-deficient parasites. Prior to beginning the TAILS protocol, reciprocally labeled SILAC proteins were isolated from WT or Δ*asp5* parasites and mixed in equal concentrations (Fig. 1C). The SILAC-TAILS samples were subjected to high pH fractionation to decrease sample complexity, resulting in 12 total fractions per SILAC pair. For dimethyl labeling, proteins were extracted from WT or *Δasp5* parasites, reciprocally blocked with either light (normal) or deuterated (heavy) formaldehyde, and then mixed and processed together for the remainder of the protocol (Fig. 1C). All samples were then desalted and subsequently run on the mass spectrometer to determine the identity of peptides. Relative peptide quantification with heavy dimethylation and SILAC-based strategies were performed in MS1 mode while area-under-the-curve measurements were performed for label-free quantitation. The N termini in the SILAC and heavy dimethyl experiments were enriched by using the TAILS method, while the HYTANE method was used for the label-free quantitative proteomics experiments (Fig. 1C).

**FIG 2:**
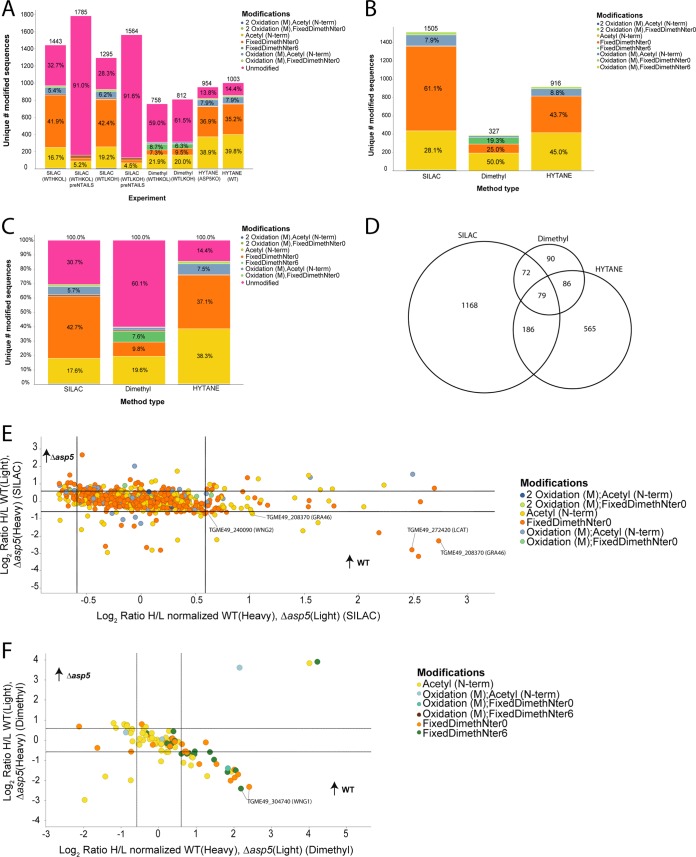
Comparison of shotgun-like pre-TAILS versus TAILS N-terminomics on *Toxoplasma* peptides. (A) Greater than 7-fold enrichment for N-terminal modified peptides was achieved in the SILAC samples compared to the pre-TAILS analyses. (B) Unravelling the features of blocked N termini. The vast majority of blocked N termini are naturally acetylated in the dimethyl and HYTANE samples or experimentally dimethylated during TAILS in the case of the SILAC samples. (C) Overall, the HYTANE method had the lowest proportion of unmodified peptides following TAILS enrichment compared with the overall lab method, which uses the HPG-ALD polymer. (D) We identified 79 modified peptides common to all three experiments, with the majority of peptides identified uniquely to the SILAC or HYTANE experiments. (E) Log-log plot representing the modified peptides with reciprocal protein expression from the SILAC-TAILS experiments. Peptides were deemed significantly differentially enriched in the WT sample if their log_2_ ratio was above 0.59 (equivalent to 1.5-fold). (F) Log-log plot representing the modified peptides with reciprocal protein expression from the heavy demethylation-TAILS experiments. Peptides were deemed significantly differentially enriched in the WT sample if their log_2_ ratio was above 0.59 (equivalent to 1.5-fold). WTHKOL: Wild type (WT) samples labelled with heavy amino acids (SILAC) or deuterated formaldehyde (Dimethyl), Δ*asp5* (KO) samples labelled with light counterparts. WTLKOH: Δ*asp5* (KO) samples labelled with heavy amino acids (SILAC) or deuterated formaldehyde (Dimethyl), WT samples labelled with light counterparts. FixedDimethNter0/6: Refers to whether the N-terminus was blocked with unlabelled (0) or heavy (6, deuterated) formaldehyde.

First, we verified N-terminal enrichment using the HPG-ALD synthetic polymer previously developed for the TAILS protocol (40) (Fig. 2A). In pre-TAILS SILAC samples, we found that only ∼9% of all identified *Toxoplasma* peptides matched N-terminally blocked peptides. However, upon TAILS enrichment, the blocked peptides consisted of ∼70% of the sample, reflecting a 7-fold N-terminal enrichment. The bulk of these modified peptides were experimentally dimethylated or naturally acetylated. Overall, we identified 2,246 N-terminal peptides across the three experiments with the majority identified by SILAC-TAILS (1,505 peptides), followed by the HYTANE strategy (916 peptides) and heavy dimethylation-TAILS (327 peptides). The majority of modified peptides in the HYTANE and dimethylation-TAILS experiments were acetylated, representing natural N termini, while the majority of peptides in the SILAC-TAILS were experimentally dimethylated (Fig. 2B). TAILS should deplete tryptic peptides indiscriminately, and therefore, it is not understood why there is variation in the identity of blocked peptides between these depletion methods. Using an alternative method for N-terminal enrichment (HYTANE), we identified a substantial improvement in the number of modified N termini with less than 15% being unmodified (Fig. 2C). Comparison of the data sets revealed 79 peptides with modified N termini that were common to each of the three quantitative proteomics experiments (Fig. 2D). Collectively, SILAC-TAILS identified the majority of N-terminal peptides; however, each approach revealed novel peptides that were not found in the other experiments, demonstrating the importance of employing multiple N-terminome methodologies.

This new TAILS data set gave us the opportunity to analyze the robustness of the current *Toxoplasma* gene annotation available on ToxoDB (toxodb.org). We found that out of the total number of unique and nonoverlapping peptides (1,911), 190 mapped precisely to the predicted start codon of the current annotation, while another 242 equated to position 2 of the predicted protein sequence, suggesting aminopeptidase activity in *Toxoplasma*. Together, these support the existing annotation of these genes. An additional 130 TAILS peptides were found at an alternative downstream methionine (Met) or subsequent amino acid, suggesting that these gene models should be reviewed. An additional 317 peptides map to other amino acids, not Met or amino acid +1, suggesting that these are internal proteolytic cleavage events. A deeper analysis of our TAILS data in relation to *Toxoplasma* gene annotation can be found in Text S1 and Table S1 in the supplemental material. Together, this demonstrates how TAILS data can be used to confirm and reannotate gene models in *Toxoplasma*.

10.1128/mBio.01796-18.6 TEXT S1 Supplemental results and methods. Download Text S1, DOCX file, 0.1 MB. Copyright © 2018 Coffey et al. 2018 Coffey et al. This content is distributed under the terms of the Creative Commons Attribution 4.0 International license.

10.1128/mBio.01796-18.7 TABLE S1 Analysis of TAILS data in relation to gene annotation on ToxoDB. Peptides identified across all experiments were filtered to identify only unique hits and identify the most upstream peptide for each gene. TAILS peptide position was then assessed relative to the predicted protein sequence (start codon), including those peptides that started with a Met or those that started at +1, others that were likely derived from nondepleted internal peptides, or those that likely represent internal protease cleavage positions. Each of these categories were then assessed for the upstream DNA sequence, thought to be indicative of start codons in *Toxoplasma*, whether or not these genes contain signal peptides, and comments on what these groupings suggest for future gene annotation. Download Table S1, PDF file, 0.1 MB. Copyright © 2018 Coffey et al. 2018 Coffey et al. This content is distributed under the terms of the Creative Commons Attribution 4.0 International license.

We then interrogated differences in the abundance of N-terminal peptides from WT and *Δasp5* tachyzoites across all methodologies. Here, we found that the SILAC-based peptide quantitation revealed 51 *Toxoplasma* peptides with modified N termini that were significantly differentially abundant between the WT and *Δasp5* samples (Table S2), including three novel ASP5 substrates—TGME49_272420 (LCAT), TGME49_208370 (GRA46), and TGME49_240090 (WNG2, previously annotated as ROP34; Fig. 2E). Differential dimethylation-TAILS revealed a total of 26 *Toxoplasma* peptides with modified N termini that were significantly differentially abundant between the WT and *Δasp5* samples (Table S2), including TGME49_304740 (WNG1, previously annotated as ROP35) (Fig. 2F). Label-free quantitative proteomics analysis revealed a number of modified peptides uniquely present in the WT samples, including TGME49_228170 (GRA44) and WNG2 (ROP34) (Table 1 and Table S2).

**TABLE 1:** List of peptides found in the combined TAILS and HYTANE data sets and subsequently validated as ASP5 substrates^*a*^

Gene name	Gene ID (TGME49_)	Sequence preceding (and location)	Peptide found	Up in WT or Δ*asp5*	Expt found	Ratio of WT/Δ*asp5* or if HYTANE, no. of samples detected in
Genes found following RRL (validated)						
LCAT	272420	RRL^476-478^	(fi)DAVLTDEVGGPESGAR	WT	SILAC (TAILS)	5.61/0.14
GRA46	208370	RRL^894-896^	(fi)LSSSAILTGQQIGTYR	WT	SILAC (TAILS)	6.65/0.21
GRA46	208370	RRL^894-896^	(n)LSSSAILTGQQIGTYR	WT	LFQ (HYTANE)	8/9 WT, 0/9 *Δasp5*
GRA44 (IMC2A)	228170	RRL^83-85^	(Ac)SGIIKTLVLWDPVQR	WT	LFQ (HYTANE)	4/9 WT, 0/9 *Δasp5*
WNG2 (ROP34)	240090	RRL^109-111^	(n)DSLIPGFLKR	WT	LFQ (HYTANE)	6/9 WT, 0/9 *Δasp5*
IST	240060	RRL^137-139^	_(ac)AAEGGSESEDEQGVAR	No ratio^*b*^	Dimethyl (TAILS)	Unable to determine*

Genes found following RRL (not validated)						
Hypothetical	233695	RRL^115-117^	QAGVYFSEEDR	WT	LFQ (HYTANE)	2/9 WT, 0/9 *Δasp5*
Hypothetical	297890	RRL^183-185^	(n)TTLASTLSLSR	WT	LFQ (HYTANE)	2/9 WT, 0/9 *Δasp5*
Hypothetical (zinc finger-containing)	248450	RRL^345-347^	(Ac)YAPGASVVESPVFGTPPSR	WT	LFQ (HYTANE)	1/9 WT, 0/9 *Δasp5*
Hypothetical	286530	RRL^27-29^ SP-cleaved^*c*^	(n)MFAAAPLQSFSVTNKQFHPE- GLEAQAPRPHQGLDMR	Neither	LFQ (HYTANE)	4/9 WT, 2/9 *Δasp5*

Genes found following predicted signal peptide cleavage site						
WNG2 (ROP34)	240090	AVA^62-64^ (SP cleaved)	(n)AHAEHPEDSATNFLFSFAENS- LANREPPEDSAARPSSR	*Δasp5*	LFQ (HYTANE)	0/9 WT, 7/9 *Δasp5*
WNG2 (ROP34)	240090	AVA^61-64^ (SP cleaved)	(fi)AHAEHPEDSATNFLFSFAE- NSLANR	Neither	SILAC (TAILS)	WTH/KOL 1.49/0.67 (not significant)
WNG1 (ROP35)	304740	AGA^68-70^ (SP cleaved?)^*d*^	(Ac)TVAAPQVETGPLLSVR	WT	Dimethyl (TAILS)	WT light dimethyl 5.31/0.18
						WT heavy dimethyl 4.17/0.81

a Locations of the peptide and three preceding amino acids were obtained from Toxodb.org v34. (fi), fixed dimethylation from SILAC experiments; (n), fixed dimethylation in HYTANE experiment; (Ac), acetylation occurring within parasites and/or host cell. The HYTANE experiment did not use differential labeling, so we could not directly compare ratios between WT versus *Δasp5*; therefore, results are displayed as number of samples the peptide was detected in per condition (*n* = 9) (3 independent biological samples, performed in triplicate, with the HYTANE procedure performed once to reduce variation).

b We were unable to determine which samples (WT or *Δasp5*) the RRL-cleaved peptide [(ac)AAEGGSESEDEQGVAR] originated from, as this was found only in the dimethyl experiment and contains no differential heavy/light dimethylation, as the N terminus is blocked and there are no lysine residues.

c Peptide for TGME49_286530 found in both WT and *Δasp5* parasites and maps within predicted SP, suggesting that this processing is mediated by signal peptidase.

d We have annotated the peptide mapping to WNG1 [(Ac)TVAAPQVETGPLLSVR] as potentially SP cleaved; however, SignalP 4.1 (58) does not recognize a signal peptide within this protein. This annotation is based on the peptide being N acetylated, a modification observed predominantly at the initiator methionine, SP cleavage site, and the ASP5 cleavage site. Spectra for HYTANE peptides can be found in Fig. S5 in the supplemental material.

10.1128/mBio.01796-18.8 TABLE S2 Full list of peptides identified in TAILS experiments and immunoprecipitations. The SILAC-TAILS tab shows a summary of the *Toxoplasma* N-terminally modified peptides deemed significantly differentially expressed (1.5-fold or greater) between the WT and Δ*asp5* parasotes with reciprocal ratios [i.e., WT(heavy)/Δ*asp5*(light) ratio of >1.5 and a WT(light)/Δ*asp5*(heavy) ratio of <0.58]. The corresponding peptide sequences and modifications are listed, including the raw file the peptide was identified in. The peptide charge, mass, and score (derived from MaxQuant) are also listed. The DiMethyl-TAILS tab shows a summary of the *Toxoplasma* N-terminally modified peptides deemed significantly differentially expressed (1.5-fold or greater) between the WT and Δ*asp5* with reciprocal ratios [i.e., WT(heavy)/Δ*asp5*(light) ratio of >1.5 and a WT(light)/Δ*asp5*(heavy) ratio of <0.58]. The corresponding peptide sequences and modifications are listed, including the raw file the peptide was identified in. The peptide charge, mass, and score (derived from MaxQuant) are also listed. The HYTANE tab shows a summary of the *Toxoplasma* N-terminally modified peptides with the preceding RRL sequence deemed significantly differentially abundant between the WT and Δ*asp5*. The corresponding peptide sequences and modifications are listed, including the raw file the peptide was identified in. The All peptides tab shows a summary of all the modified peptides identified across all experiments in this study. The IP tabs show accession numbers and gene names of proteins determined to be statistically enriched (up or down) following comparison of the immunoprecipitation (IP) results of the bait proteins listed. Proteins listed in red are highlighted on the plot in Fig. S2. Download Table S2, XLSB file, 3.9 MB. Copyright © 2018 Coffey et al. 2018 Coffey et al. This content is distributed under the terms of the Creative Commons Attribution 4.0 International license.

In total, we identified more than 2,000 unique N-terminal peptides by the three methodologies. Many of these peptides were not significantly different between WT and *Δasp5* parasites, having arisen from natural N termini (both exposed and acetylated) as well as from other protease cleavage events within parasites. However, each methodology also revealed unique peptides that mapped directly after an RRL motif, enabling the identification of likely novel ASP5 substrates.

### The PVM protein LCAT is processed by ASP5.

**FIG 3:**
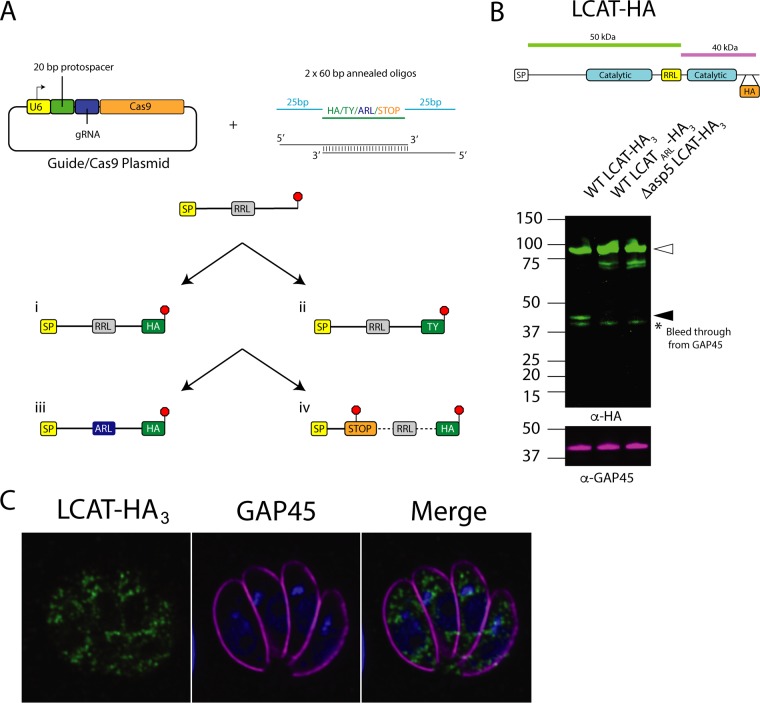
Methodology for validation of ASP5 substrates. (A) Schematic of endogenous tagging strategy. U6, U6 promoter; protospacer, 20- to 21-bp sequence used to direct Cas9 to cut within the parasite genome; gRNA, guide RNA; Cas9, enzyme that cuts DNA; SP, signal peptide; RRL, arginine-arginine-leucine, site of cleavage by ASP5; HA, hemagglutinin tag; Ty, Ty tag; red stop signal, endogenous stop codon or introduced stop codon/frameshift mutation; oligos, oligonucleotides; ARL, mutation of first arginine to alanine. (B) Immunoblot of LCAT-HA_3_ (TGGT1_272420) using the LI-COR Odyssey imager, anti-HA (αHA) and αGAP45 antibodies used. Open arrowheads indicate predicted full-length species; closed arrowheads indicate predicted ASP5-cleaved species. (C) IFA of LCAT-HA_3_-expressing parasites. HA and GAP45 antibodies were used. DNA was stained using DAPI (LCAT [TGGT1_272420]).

We sought to validate newly identified proteins as ASP5 substrates. To do this, we endogenously tagged candidate proteins in both wild-type and Δ*asp5* tachyzoites and then mutated the putative RRL cleavage site to look for differential processing within parasites. For tagging and mutagenesis of almost all proteins, we designed Cas9-targeting guides against the gene of interest and cotransfected these with two annealed ∼60-bp oligonucleotides to introduce either an epitope tag, mutate RRL to ARL (RRL→ARL), or disrupt the gene (Fig. 3A). We first chose to investigate a recently discovered dense granule protein LCAT (TGME49_272420) that is secreted to the PVM, as we identified an N-terminal peptide found exclusively in WT parasites with the sequence (dimethyl)-DAVLTDEVGGPESGAR (Table 1), which mapped to a location directly C terminally of an RRL sequence within this protein (42). LCAT is processed into two fragments by an unknown protease within the inserted element (IE) that separates the catalytic residues of the enzyme (42). As the TAILS peptide mapped to directly after an RRL within this IE, we tagged endogenous LCAT and observed an ∼90-kDa “full-length” species and an ∼40-kDa processed form (Fig. 3B). We then tagged LCAT in *Δasp5* parasites and primarily observed the larger-molecular-weight species (Fig. 3B), with a doublet band at ∼60 kDa that may be a degradation product or result from a subsequent processing event. To confirm that the loss of the ∼40-kDa species was at the TEXEL sequence, we then swapped the endogenous RRL to ARL (schematic in Fig. 3A) and observed only the larger species in WT LCAT_ARL_-HA. Together, this strongly suggests that LCAT is processed at the TEXEL motif identified by TAILS (Table 1).

It is important to note that by immunofluorescence assays (IFA), we could detect LCAT only within punctate structures within parasites, possibly the dense granules (Fig. 3C), but not at the PVM as has previously been reported (42). The absence of signal at the PVM suggests that the introduction of the 3xHA tags or replacement of the endogenous 3′ untranslated region (3′ UTR) with the dihydrofolate reductase (DHFR) 3′ UTR interfered with trafficking of this enzyme (Fig. 3C). To overcome this, we attempted to introduce a single hemagglutinin (HA) tag into the endogenous locus as this has been shown not to affect trafficking (42). However, despite several attempts, we were unable to introduce this single HA tag into the endogenous LCAT locus without positive selection, and therefore, we were unable to assess any differences in localization in LCAT between WT and *Δasp5* parasites (data not shown).

### N-terminal peptide enrichment identifies novel dense granule proteins as ASP5 substrates.

**FIG 4:**
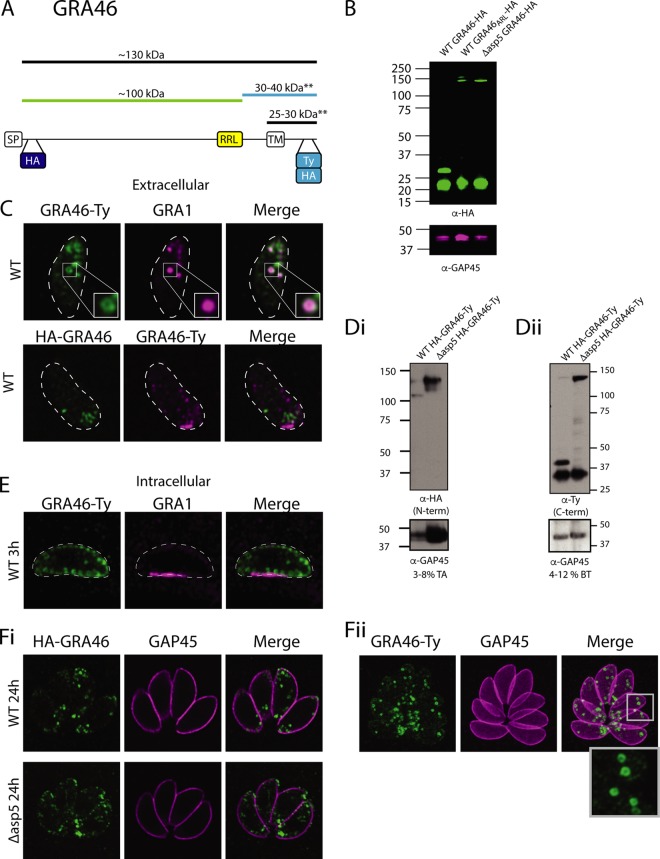
GRA46 is a novel ASP5 substrate. (A) Schematic of TGME49_208370 (GRA46) with a HA tag inserted after the signal peptide cleavage site, the position of the predicted TEXEL (RRL), predicted transmembrane domain (TM), and a Ty or HA tag inserted prior to the stop codon. The ** indicate the precise sizes are not known, as these species were seen to migrate at different rates when using -HA (B) and -Ty epitope tags (Dii). (B) Immunoblot using αHA antibodies (C-terminal fragment). (C) Extracellular IFAs, where the white dotted line represents the plasma membrane. (D) Western blot on HA-GRA46-Ty in WT and Δ*asp5* parasites using αHA antibodies (N-terminal fragment) (Di) and on HA-GRA46-Ty in WT and Δ*asp5* parasites using αTy antibodies (C-terminal fragment) (Dii). TA, Tris-acetate gel; BT, Bis-tris gel. (E) IFA at 3 h postinvasion. (F) IFA at 24 h postinvasion of replicating parasites using αHA antibodies (Fi) and αTy antibodies (Fii).

After validating the dense granule protein LCAT as an ASP5 substrate, we sought to investigate novel and hypothetical candidates. One peptide arising after an RRL only in WT parasites was Ac-SSSAILTGQQIGTYR (Table 1), mapping to a hypothetical protein (TGME49_208370), which we have named GRA46. This protein was chosen for further investigation, as the RRL motif maps near the C terminus of the protein prior to a predicted transmembrane domain, similar to MYR1 (Fig. 4A). GRA46 was interesting, as it is annotated on ToxoDB as “myosin heavy chain-like,” but further investigation has revealed limited homology to myosin, rendering it unlikely that this protein is a true motor protein (data not shown). To assess GRA46 as a potential ASP5 substrate, we inserted a HA tag at the C terminus in WT parasites and observed two species, one at approximately 22 kDa and another at ∼30 kDa, both well below the predicted size of ∼125 kDa (Fig. 4A and B). We then repeated this process in *Δasp5* parasites, revealing the lowermost band and a second band at ∼140 kDa, indicating the loss of a processing event, dependent on ASP5. To validate the RRL found by TAILS within this vicinity, we mutated RRL→ARL (Fig. S1A), and again observed the same processing pattern as seen in *Δasp5* parasites, strongly suggesting that GRA46 is cleaved by ASP5 in a TEXEL-dependent manner. The presence of the lowest-molecular-weight species in WT, ARL, and *Δasp5* parasites potentially results from processing at or near the predicted transmembrane (TM) domain near the C terminus of this protein by a protease other than ASP5 (Fig. 4A and B).

10.1128/mBio.01796-18.1 FIG S1 Genotyping of parasite lines generated in this study. (Ai) Genetic strategy and Sanger sequencing of GRA46 RRL→ARL. (ii) Schematic representation and PCR confirmation of Δ*gra46* parasites. (B) Genetic strategy and Sanger sequencing of WNG1_ARL_HA parasites (top) and *Δwng1* parasites (lower). (C) Genetic strategy and Sanger sequencing of WT (upper), WNG2_ARL_-HA (middle), and *Δwng2* (lower) parasites. (D) Genetic strategy and PCR validation of knockout of GRA44 confirming integration of the LoxP-DHFR cassette (right) and the WT GRA44 locus (left). (E) Genetic strategy and validation of deletion of ASP5 by Sanger sequencing. Download FIG S1, TIF file, 1.1 MB. Copyright © 2018 Coffey et al. 2018 Coffey et al. This content is distributed under the terms of the Creative Commons Attribution 4.0 International license.

As both MYR1 and LCAT are cleaved by ASP5 near the C terminus, and then the N and C termini reassociate (38, 42), we sought to determine whether the same was true for GRA46. To address this, we tagged GRA46 at the C terminus with a TY tag, followed by a HA tag shortly after the predicted signal peptidase cleavage site (Fig. 4A). To determine the subcellular localization of the two ASP5-processed sequences of GRA46, we probed extracellular parasites with anti-Ty (αTy) antibodies using superresolution microscopy and observe a spherical donut-shaped localization that encloses GRA1 staining in dense granules, suggesting that this protein resides at the periphery of the dense granules (Fig. 4C). We observe very low levels of colocalization of the N-terminal (anti-HA [αHA]) and C-terminal (αTy) fragments of GRA46 in extracellular parasites (Fig. 4C, bottom panel).

Immunoblot analysis of WT parasites expressing HA-GRA46-TY using αHA antibodies revealed two bands at approximately 130 and 100 kDa, while only the higher-molecular-weight species was present in Δ*asp5* parasites, suggesting a loss of processing (Fig. 4Di). Using αTy antibodies, the majority of this protein was detected at ∼40 kDa, while in Δ*asp5* parasites, this species was lost, and the protein remained at a higher molecular weight, consistent with the full-length protein (Fig. 4Dii). As in Fig. 4B, we observed a consistent ∼30-kDa band for GRA46 in both WT and Δ*asp5* parasites and this observation is consistent with processing around the TM domain of this protein by a protease other than ASP5 (Fig. 4Dii). It is important to note that we see a consistent size difference of the lower-molecular-weight species between GRA46-HA and HA-GRA46-Ty. We do not know why this occurs, but it may have to do with differences in the configuration of the epitope tag used.

We then proceeded with further assessment of the localization of GRA46. To determine the location of this protein during early infection, parasites were fixed at 3 h postinvasion, which revealed the secretion of GRA1 into the PV, but GRA46 appeared to remain associated with the dense granules (Fig. 4E). At 24 h postinvasion, the N-terminal processed species of GRA46 in WT and *Δasp5* parasites localized to discrete puncta within the parasites, notably at the anterior and posterior ends of the tachyzoites (Fig. 4Fi). At this time point, the C-terminal fragment also localized to similar spherical structures within the parasites, potentially dense granules (Fig. 4Fii). We could detect no difference in localization of the C-terminal fragment between WT and *Δasp5* parasites.

Together, these data reveal that GRA46 is processed by ASP5 near the C terminus and that it is a dense granule protein. The TEXEL of GRA46 maps to the C-terminal end of this protein; however, unlike MYR1 and LCAT that are cleaved by ASP5 within a similar region, the N- and C-terminal fragments of GRA46 do not appear to reassociate. We were unable to detect GRA46 within the host cell, suggesting that it is not exported, similar to MYR1 and LCAT.

### Two WNG kinases are substrates of ASP5.

Further investigation of our proteomic data set revealed two proteins that were previously annotated as rhoptry kinases through their similarity to ROP16, ROP18, and several other predicted kinases (Table S2) (43). Here we present ROP35 (TGME49_304740) and ROP34 (TGME49_240090), herein named WNG1 and WNG2, respectively, for reasons outlined below, as novel ASP5 substrates. WNG1 was identified in the TAILS screen due to increased levels of acetylated SP-cleaved peptide within Δ*asp*5 parasites (Table 1) that mapped upstream of a TEXEL motif, suggesting that the SP cleavage site is the N terminus in *Δasp5* parasites.

**FIG 5:**
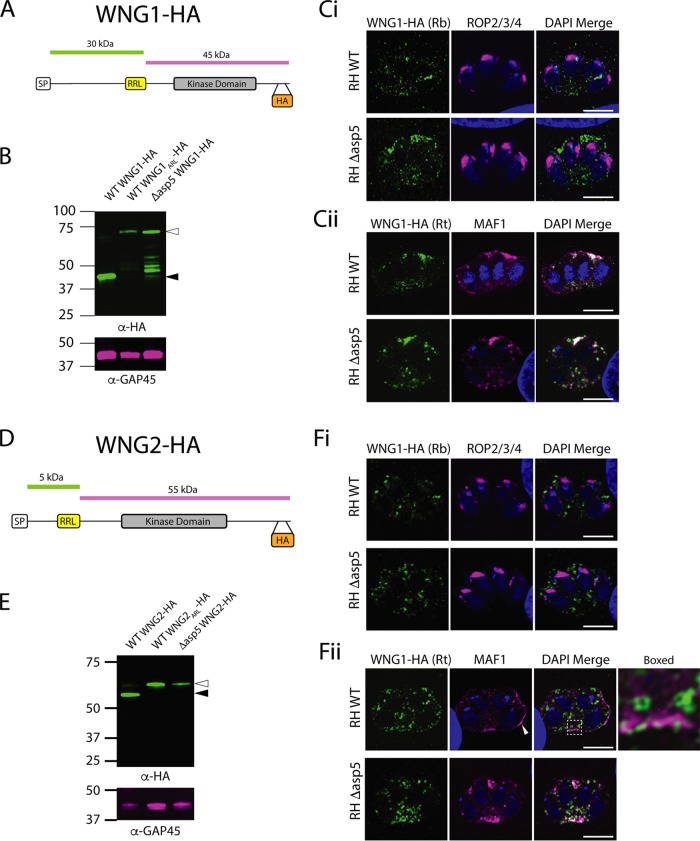
WNG1 and WNG2 are ASP5 substrates that localize to the host-parasite interface. (A) Schematic of WNG1. (B) Immunoblot using the LI-COR Odyssey imager. Open arrowheads indicate predicted full-length species; closed arrowheads indicate predicted ASP5-cleaved species. (C) IFAs at 24 h postinvasion with rabbit (Rb) αHA antibodies and the rhoptry marker αROP2/3/4 (Ci) and IFAs with rat (Rt) αHA antibodies and the dense granule and PVM marker MAF1 (Cii). (D) Schematic of WNG2. (E) Immunoblot using the LI-COR Odyssey imager. Open arrowheads indicate predicted full-length species; closed arrowheads indicate ASP5-cleaved species. (F) IFAs at 24 h postinvasion with rabbit (Rb) αHA antibodies and the rhoptry marker αROP2/3/4 (Fi) and IFAs with rat (Rt) αHA antibodies and the dense granule and PVM marker MAF1 (Fii).

To determine the fate of WNG1, we epitope tagged this protein at the C terminus and monitored its maturation by Western blot (Fig. 5A and B). We observed that WNG1 expressed by WT parasites was present at ∼42 kDa, which is a smaller size than predicted by the full amino acid sequence (Fig. 5A). In contrast, epitope tagging in *Δasp5* parasites revealed that WNG1 was present at a higher molecular weight, suggesting that it is indeed processed by ASP5 (Fig. 5B). To assess whether WNG1 is processed within the predicted TEXEL motif, we mutated the endogenous RRL→ARL (Fig. S1B), and indeed, observed an accumulation of the higher-molecular-weight species in these WNG1_ARL_-HA parasites (Fig. 5B).

We then investigated the localization of WNG1 by IFA and found no colocalization with the αROP2/3/4 marker in intracellular parasites, suggesting that this predicted kinase is not a true rhoptry protein (Fig. 5Ci). The low level of HA signal observed using rabbit αHA antibodies in WT WNG1-HA-expressing parasites (Fig. 5Ci, upper panel) was more pronounced in *Δasp5* parasites (lower panel), suggesting that loss of ASP5 leads to the accumulation of WNG1 within localized areas in the PV, a phenomenon observed for several dense granule proteins following deletion of ASP5 (21, 23) (Fig. 5Ci). WT:WNG1-HA was primarily found within the vacuole and exhibited partial colocalization with the PV/PVM marker MAF1 (Fig. 5Cii), as did *Δasp5*:WNG1-HA, suggesting that WNG1 is a dense granule protein.

We then investigated WNG2 and its dependence on ASP5 for proteolytic maturation. Our N-terminome data identified a peptide that mapped to WNG2, resulting from cleavage after an RRL (dimethyl-DSLIPGFLKR) was observed in WT but not in Δ*asp*5 parasites (Table 1). To confirm whether WNG2 is a true ASP5 substrate, we inserted a HA tag into the endogenous locus just before the stop codon and observed the dominant species at ∼55 kDa, despite the predicted molecular weight of ∼62 kDa. In contrast, in parasites lacking ASP5, and those expressing the mutated RRL→ARL (WNG2_ARL_-HA; Fig. S1D), this protein migrated at approximately 60 kDa, strongly suggesting ASP5-dependent maturation (Fig. 5E).

We then investigated the localization of WNG2 by IFA. As with WNG1, WNG2 did not colocalize with the rhoptry marker αROP2/3/4 (Fig. 5Fi). When probing with antibodies against the PV/PVM protein MAF1 (Fig. 5Fii), a small proportion of WNG2-HA expressed by WT parasites colocalized at the PVM (see closed arrowhead); however, most was present within the parasites within the donut-shaped spheres (boxed area), similar to that described for GRA46, suggestive of dense granules (Fig. 4C). In *Δasp5* parasites, MAF1 loses a significant proportion of its PVM localization, as has been previously reported (21), and the WNG2-HA signal in *Δasp5* parasites overlaps with the MAF1 signal detected in the PV space. Our data therefore strongly suggest that these kinases are unlikely to represent bona fide rhoptry proteins, and we have therefore renamed these proteins WNG kinases, short for “With No Gly loop” as they do not contain the characteristic glycine-rich loop of other kinases. As ROP35 is the most conserved across Coccidia, we renamed ROP35 WNG1 and renamed ROP34 WNG2. It will be interesting in the future to determine whether the absence of the glycine-rich loop region renders these kinases inactive or whether they still retain enzymatic activity (43).

### ASP5 matures the dense granule phosphatase GRA44 and a novel dense granule protein.

Our N-terminome analyses discovered a peptide resulting from cleavage after an RRL within the protein annotated as inner membrane complex protein 2A (IMC2A, TGME49_228170). For reasons outlined below, we renamed IMC2A GRA44.

**FIG 6:**
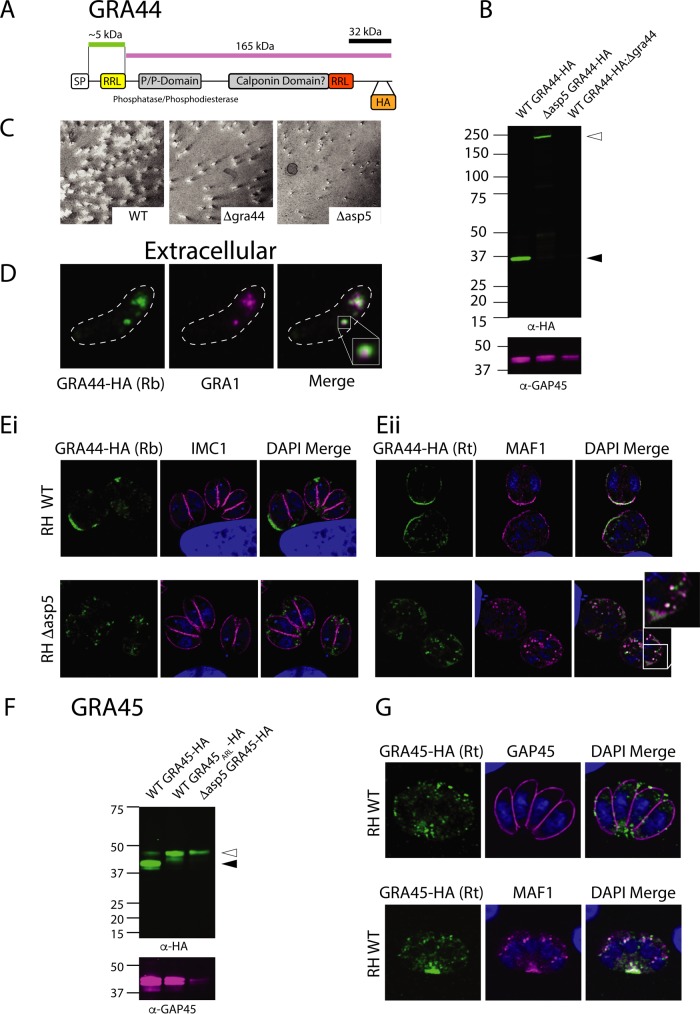
GRA44 is matured by ASP5 and localizes to the PVM. (A) Schematic of GRA44 based on the TGGT1_228170 sequence. (B) Immunoblot using the LI-COR Odyssey imager and using αHA and αGAP45 antibodies. Open arrowheads indicate the expected size of full-length species; closed arrowheads indicate predicted ASP5-cleaved species. (C) Plaque assay at 9 days depicting plaque sizes for WT (RH*Δku80Δhx*), *Δgra44* (RH*Δku80ΔhxΔgra44*), and *Δasp5* (RH*Δku80ΔhxΔasp5*) parasites. (D) IFA of an extracellular tachyzoite depicting WT GRA44–HA and the dense granule marker GRA1. (E) IFAs of intracellular WT- and *Δasp5*-GRA44-HA parasites with the IMC marker IMC1 (Ei) and the dense granule marker MAF1 (Eii). (F) Immunoblot using the LI-COR Odyssey imager and using αHA and αGAP45 antibodies. Open arrowheads indicate the predicted full-length species; closed arrowheads indicate predicted ASP5-cleaved species. (G) IFA of WT intracellular parasites expressing GRA45-HA (green) with GAP45 (top panel) and MAF1 (bottom panel).

To investigate the maturation of GRA44 by ASP5 we introduced a HA tag adjacent to the endogenous stop codon in WT parasites (Fig. 6A). We observed a species of ∼37 kDa, shorter than the predicted ∼185-kDa size of the full-length protein (Fig. 6A and B). In contrast, this protein migrated close to 200 kDa in *Δasp5* parasites, suggesting the loss of one or more processing events within these parasites. The RRL-cleaved peptide of GRA44, found in both the dimethyl and HYTANE experiments, was observed only in WT parasites and maps near the predicted SP cleavage site. However, we were unable to mutate this endogenous RRL→ARL despite several attempts, suggesting a fitness cost to the parasites (data not shown). Processing at this N-terminal RRL could not account for the ∼37-kDa species observed in WT parasites, suggesting that GRA44 is processed at least twice. Indeed, GRA44 contains a second RRL approximately 32 kDa from the C terminus (Fig. 6A) which may be processed by ASP5. This is, however, yet to be proven, as we were unable to make point mutations in this region and we did not find TAILS peptides in our data set corresponding to this putative cleavage site.

To investigate the importance of GRA44 on parasite growth, we generated a knockout line by integration of CAT selectable marker (Fig. S1D), which led to the ablation of HA signal by Western blotting (Fig. 6B; longer exposure in Fig. S2). Parasites lacking GRA44 (*Δgra44*) formed smaller plaques than WT parasites, yet the defect was not as severe as that observed for *Δasp5* parasites, despite having a lower CRISPR fitness score (44) (Fig. 6C). In extracellular parasites, GRA44–HA signal enclosed a subset of GRA1-containing dense granules, similar to GRA46 (Fig. 6D and 4C). Immunofluorescence assays demonstrate a lack of colocalization with IMC1 in intracellular parasites, suggesting that this protein is not part of the IMC, as its previous annotation of IMC2A suggests, and instead localizes within the PV and PVM in WT parasites (Fig. 6Ei and ii). In Δ*asp5* parasites, this protein is observed primarily between parasites in the PV instead of the PVM, similar to MAF1 (Fig. 6E), as is common for dense granule proteins after deletion of ASP5 (21–23).

10.1128/mBio.01796-18.2 FIG S2 Identification of GRA44-, GRA46-, and WNG2-interacting proteins by quantitative mass spectrometry. Volcano plots illustrating the log_2_ protein ratios of immunoprecipitated proteins and the significance of the protein changes (−log_10_ *P* value BH corrected). Proteins were deemed differentially expressed if the log_2_ fold change in protein expression was greater than 2-fold (red) or 4-fold (green) and a −log_10_ *P* value of ≥1.3, equivalent to a *P* value of ≤0.05. Pairwise comparisons were made with the various HA-tagged bait proteins, including GRA44-HA/GRA46-HA (A), GRA44-HA/WNG2-HA (B), and WNG2-HA/GRA46-HA (C). Download FIG S2, TIF file, 1.2 MB. Copyright © 2018 Coffey et al. 2018 Coffey et al. This content is distributed under the terms of the Creative Commons Attribution 4.0 International license.

To elucidate further the mechanisms by which the novel ASP5 substrates operate, we performed immunoprecipitations (IPs) to assess whether any act within larger complexes. To address this, we infected human foreskin fibroblasts (HFFs) at a multiplicity of infection (MOI) of 5 with GRA46-HA, GRA44-HA, and WNG2-HA parasites, then harvested from large vacuoles at 36 h postinfection, lysed, and then pulled down these proteins using αHA antibodies. IPs were performed in triplicate, and protein eluates were subjected to mass spectrometry analysis where protein expression changes were quantified using a custom label-free pipeline. Each of the pulldowns enriched the bait (Fig. S3), while also enriching for several other dense granule proteins. TGME49_316250 (GRA45) was highly enriched in GRA44-HA and WNG2-HA pulldowns compared to GRA46-HA (Fig. S3 and Table S2) and was chosen for further validation, as it contains a signal peptide and a putative TEXEL motif.

10.1128/mBio.01796-18.3 FIG S3 GRA44-HA immunoblot, as in Fig. 6B, overexposed. Arrows depict predicted sizes of processed (black) and unprocessed (white) fragments. Download FIG S3, TIF file, 1.4 MB. Copyright © 2018 Coffey et al. 2018 Coffey et al. This content is distributed under the terms of the Creative Commons Attribution 4.0 International license.

To investigate TGME49_316250, which we herein rename GRA45, we epitope tagged this protein just before the predicted stop codon. The dominant molecular weight species observed in WT parasites was approximately 42 kDa, with a fainter band at ∼47 kDa (Fig. 6F). Deletion of ASP5 or mutation of the RRL→ARL resulted in the loss of the lower migrating band, suggesting this protein is indeed processed by ASP5 (Fig. 6F). GRA45-HA was observed in punctate structures within parasites and the PV space, primarily at the posterior of parasites (Fig. 6G), somewhat overlapping with the dense granule protein MAF1. Similar localization was observed in *Δasp5* parasites (not shown), suggesting this novel ASP5 substrate is a dense granule protein.

### WNG2 is important for acute-stage virulence.

**FIG 7:**
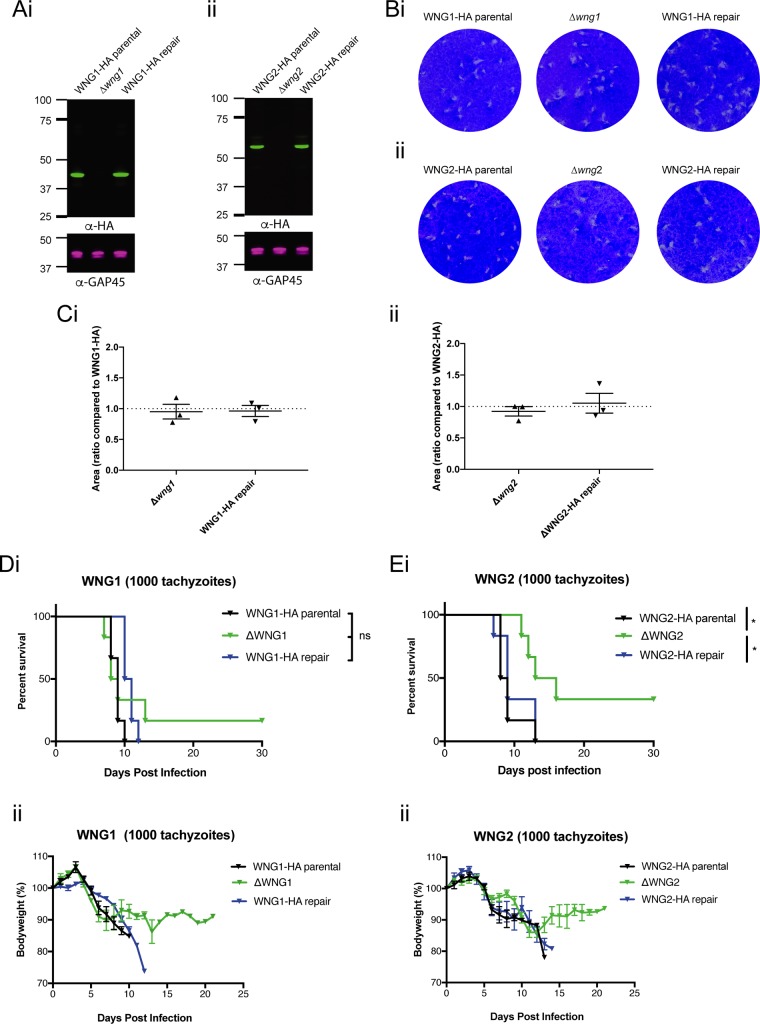
WNG2, but not WNG1, is important for acute virulence. (A) Western blot confirming genetic disruption and repair of WNG1 (Ai) and WNG2 (Aii) in Pru*Δku80Δhxgprt* parasites. Note that in longer exposures, both the full-length and ASP5-processed bands were present, as in RH parasites (Fig. 5). (B) Plaque assays of WNG-HA parental, Δ*wng1*, and WNG1-HA repair (Bi) and WNG2-HA parental, Δ*wng2*, WNG-HA repair (Bii). (C) Quantification of plaque area in Δ*wng1*, WNG1-HA repair (Ci) and Δ*wng2*, WNG2-HA (Cii) repair relative to parental (WNG1-HA or WNG2-HA) controls, respectively. (D) Kaplan-Meier survival curves of C57BL/6 mice infected with 1,000 WNG1 transgenic tachyzoites as indicated (Di) and pooled body weight (ii). (E) Kaplan-Meier survival curves of C57BL/6 mice infected with 1,000 WNG2 transgenic tachyzoites as indicated (Ei) and pooled body weight (Eii). Survival curve comparisons were assessed using the log rank (Mantel-Cox) test, and significance was assessed following correction for the Bonferroni threshold when performing multiple comparisons. Statistical significance is indicated as follows: *, *P* value of <0.05; ns, nonsignificant.

ROP kinases are important virulence factors (4–12); therefore, we wanted to determine whether the WNG kinases also played important roles *in vivo*. We therefore generated knockouts of WNG1 and WNG2 (Fig. S1B and C) in Pru*Δku80Δhx* parasites and confirmed loss of expression by Western blotting (Fig. 7Ai and ii). The *wng1* and *wng2* loci containing these deletions were then complemented through transfection with a unique CRISPR/Cas9 guide coupled with an HDR template derived from the WT sequence of the gene, which led to the reinstatement of expression of these kinases (WNG1/2-HA repair). To determine whether there was any growth defect *in vitro* of Δ*wng1* and Δ*wng2* parasites, we performed plaque assays (Fig. 7Bi and ii) and quantitated the size of zones of clearance (Fig. 7Ci and ii). In doing so, we found no difference between parental lines and either Δ*wng1* and Δ*wng2* parasites or their corresponding repaired lines.

We then sought to determine whether WNG1 and WNG2 are important for parasite virulence during acute infection. For purposes of comparison, we first generated a knockout of ASP5 in a type II Pru background (Fig. S1E). To confirm that the loss of ASP5 in Pru also leads to attenuation, we injected six C57/BL6 mice with 5,000 or 50,000 parasites and found that all these mice survived the challenge compared to infection with the Pru*Δku80* parent, all of which succumbed by day 10 (Fig. S4Ai). Further, mice infected with *Δasp5* parasites retained their initial body weight (Fig. S4Aii), despite exhibiting bloating of the abdomen, a common response to infection. We then injected six C57/BL6 mice with 1,000 Pru*Δku80* WT, *Δwng1*, and *Δwng2* parasites and their corresponding repaired lines and monitored infection (Fig. 7D and E). Here we found that mice infected with Δ*wng1* tachyzoites succumbed at approximately the same time as their parental and repair counterparts; however, one mouse inoculated with the knockout survived (Fig. 7Di and ii). In contrast, loss of WNG2 led to a measurable and statistically significant change in time to death compared to parental and repair lines (Fig. 7Ei). Furthermore, the remaining mice infected with *Δwng2* parasites regained body weight over the experimental time frame (Fig. 7Eii). We also infected mice with 500 tachyzoites (Fig. S4B and C), which largely confirms our findings with infection with the higher dose. Furthermore, for both TEXEL proteins assessed in this experiment, there was no statistical difference in survival time or weight loss for mice infected with WT or RRL→ARL mutants, suggesting that for these nonexported ASP5 substrates, efficient processing by ASP5 is not essential for their function during murine infection (data not shown). All surviving mice were seropositive for exposure to *Toxoplasma* (data not shown).

10.1128/mBio.01796-18.4 FIG S4 Virulence of PruΔ*asp5*, Δ*wng1*, and Δ*wng2* parasites. (A) Mice were infected with either 5 × 10^3^ or 5 × 10^4^ WT or Δ*asp5* tachyzoites intraperitoneally, and virulence was measured by survival over time (i) and body weight (as a percentage of starting weight) (ii). (B) Five hundred parental (WNG1-HA), Δ*wng1*, and WNG1-HA-repaired parasites were administered intraperitoneally, and virulence was measured by survival over time (i) and body weight (ii). (C) Five hundred parental (WNG2-HA), Δ*wng2*, and WNG2-HA-repaired parasites were administered intraperitoneally, and virulence was measured by survival over time (i) and body weight (ii). Download FIG S4, TIF file, 1.2 MB. Copyright © 2018 Coffey et al. 2018 Coffey et al. This content is distributed under the terms of the Creative Commons Attribution 4.0 International license.

10.1128/mBio.01796-18.5 FIG S5 MS/MS fragmentation spectra of LFQ (HYTANE) peptides presented in Table 1. Download FIG S5, PDF file, 0.5 MB. Copyright © 2018 Coffey et al. 2018 Coffey et al. This content is distributed under the terms of the Creative Commons Attribution 4.0 International license.

## DISCUSSION

*Toxoplasma* uses a repertoire of secreted and exported proteins to exquisitely modulate the host response to infection. Initial host cell subversion is achieved through the secretion of the rhoptry proteins, notably ROP16 and ROP5/18 (4–6). Following establishment of the PV, *Toxoplasma* translocates a second wave of proteins across the PVM. These proteins include two ASP5-cleaved exported proteins, GRA16 and IST, that *Toxoplasma* employs to regulate the cell cycle and avoid clearance from cells following IFN-γ signaling, respectively. Furthermore, GRA24 does not appear to be processed by ASP5, yet its translocation across the PVM is still dependent on this protease (17, 21–23). Interestingly, the translocation of these proteins into the host cell requires the PVM protein MYR1, another ASP5 substrate. Despite the importance of this protease, only a handful of ASP5 substrates have been described thus far. To address this shortcoming and identify new *Toxoplasma* effectors and virulence factors, we have exploited the differences in N termini between WT and *Δasp5* parasites using a quantitative proteomics approach in combination with N-terminal peptide enrichment methods. We have validated several of these substrates, including confirmation of their processing by ASP5.

Here we have presented evidence that WNG1, WNG2, GRA44, GRA45, and GRA46 are processed by ASP5. Notably, immunofluorescence of each of these proteins has revealed they are at least transient components of the dense granules. It is currently unclear whether ASP5 processes substrates from other organelles, including the micronemes and the rhoptries; however, proteins from these organelles have recently been demonstrated to be processed by aspartyl protease 3 (ASP3) (45), suggesting that different proteases may function in maturation of proteins destined for different cellular compartments. Following ASP5 cleavage, we observe secretion of these newly validated substrates into the PV/PVM, with no signal detected within the host cell. However, we cannot preclude the possibility that some of these proteins are indeed translocated across the PVM but subsequently are highly diluted and could not be detected through normal immunofluorescence imaging. Indeed, the four characterized exported proteins GRA16, GRA24, GRA28, and IST all traffic to the host nucleus (15, 17–20), potentially concentrating their signal above levels that could be detected if they were distributed throughout the cytosol.

Unlike in *Plasmodium*, the TEXEL sequence in *Toxoplasma* appears not to be spatially restricted to the N terminus of the protein. The identification of new ASP5 substrates in this study confirms that the TEXEL motif can be found anywhere throughout the protein. However, in the two exported substrates, GRA16 and IST, this motif is located approximately 40 and 70 amino acids from the predicted SP cleavage site or at 13% and 16% of the protein length, respectively. As GRA44 is also processed within this approximate region, cleavage by ASP5 within this vicinity alone is unlikely to dictate translocation across the PVM. It is possible that ASP5 processing potentially liberates an export signal, such as the linear or structural sequence of amino acids revealed following cleavage, a suggestion that has also been raised for export of PEXEL proteins (29). To address this, both a nonexported protein and an exported protein should be monitored for their ability to translocate the PVM following targeted mutagenesis of the TEXEL motif and surrounding residues. This could reveal whether residues either upstream or downstream of cleavage are important for subsequent trafficking and ultimately export into the host cell.

We have demonstrated that two proteins previously predicted to be rhoptry kinases instead appear to be dense granule proteins. Importantly, both WNG1 and WNG2 contain the key kinase sequence motifs, suggesting they should be active within the dense granules and/or PV space; however, this is yet to be confirmed. WNG1 (ROP35) was recently knocked out in PruΔ*ku80* parasites as part of a wider screen to identify the role of the predicted rhoptry kinases during chronic infection (46). Interestingly, although *Δwng1* parasites retained the ability to differentiate into bradyzoites *in vitro*, parasites lacking this kinase formed approximately 75% fewer tissue cysts within the brains of infected mice, suggesting WNG1 plays a role in the development or persistence of bradyzoites (46). Kinases have already been extensively explored as effector proteins in *Toxoplasma*, ranging from the secreted ROP16 and ROP18, to the recently described (dense granule protein kinases) annotated as ROP21 and ROP27 (47). While these kinases have been characterized at the host-parasite interface, no phosphatases had been reported at this location. In light of this, a novel finding in this study was the localization of GRA44 at the PVM. This is the first example of a phosphatase that lies at the interface of the host-parasite boundary. Interestingly, GRA44 has a CRISPR score of −3.28 (44), lower than ASP5’s score of −1.45, which suggested that this protein is important for *in vitro* growth. This CRISPR score was validated as *Δgra44* parasites exhibited a growth defect *in vitro*, a phenotype that has not previously been observed for ASP5-cleaved dense granule proteins, including GRA16 and MYR1, despite parasites lacking both of these proteins displaying a substantial virulence defect *in vivo* (15, 38). Finally, GRA44 was originally predicted to localize to the IMC based on an antibody that recognized the cytoplasmic face of the IMC, whose target was named IMC2 (48). The current version (v34) of ToxoDB does not recognize a protein termed IMC2, and the sequence used to immunize mice to generate the original IMC2 antibody is no longer annotated as part of IMC2A. Importantly, despite several attempts, we were unable to genetically manipulate the RRL that precedes the predicted phosphatase/phosphodiesterase domain, suggesting this change resulted in a fitness defect in parasites. Furthermore, this region was also refractory to the introduction of a Ty epitope tag, and therefore, we were unable to directly observe the trafficking of this much larger fragment in WT parasites. However, the loss of the ∼37-kDa GRA44-HA species in Δ*asp5* parasites suggests GRA44 is indeed processed by ASP5 at the C-terminal RRL, and as the HA-tagged protein detected by IFA in these parasites contains the phosphatase/phosphodiesterase domain and similarly traffics to the PV, we posit that this is also true in WT parasites.

To assess the role of these newly described ASP5 substrates *in vivo*, we employed a mouse model to assess *Toxoplasma* infection in C57BL/6 mice. As a control, we inoculated mice with up to 50,000 Pru*Δku80Δasp5* (*Δasp5*) tachyzoites and observed complete attenuation in virulence, while mice infected with proportional numbers of WT parasites succumbed during this time period (see Fig. S4 in the supplemental material). To validate the role of ASP5 cleavage in virulence for WNG1 and WNG2, we infected mice with knockouts of these putative kinases. While we found that WNG2 contributes to tachyzoite virulence, surprisingly, we observed no significant difference in virulence for mice infected with WT WNG2-HA parasites compared to WNG2_ARL_-HA parasites (data not shown). These data suggest that at least for these nonexported proteins, efficient ASP5 processing is not required for their trafficking to the dense granules, PV and/or PVM. This however, does not discount the possibility that a small amount of protein may still be cleaved and that this is enough to fulfil its function.

It is clear that there are more ASP5 substrates than we have identified. Not all ASP5 substrates were detected in our analyses, including GRA16 and MYR1. There are several potential reasons for this, with two of the most likely being: (i) the ASP5-cleaved protein may be of low abundance in sampled parasites, and (ii) the subsequent peptides are difficult to enrich or not suitable for detection by mass spectrometry. The former point is supported by a recent study utilizing TAILS to identify substrates of ASP3 in *Toxoplasma* (45). ASP3 processes microneme and rhoptry proteins that are considered more abundant than the dense granule proteins, potentially enabling greater substrate identification. Furthermore, it could be that ASP3 substrates greatly outnumber ASP5 substrates, thus explaining the larger number of substrates detected during interrogation of ASP3. Overall, while it is clear that using two different methodologies (i.e., TAILS and HYTANE) to enrich for N-terminal peptides was beneficial (40, 41), other methodologies will need to be adopted to identify more ASP5 substrates.

In conclusion, we have used N-terminomics to identify novel substrates of the important protease ASP5 and validated a subset of these through tagging and mutagenesis of the endogenous locus within parasites. Deletion of WNG2 corresponded to a reduction in virulence during murine infection, but not to the same extent as infection with *Δasp5* parasites, suggesting that the dramatic reduction in virulence during *Δasp5* infection results from many effectors that are not trafficked correctly. Overall, our data validate the essential role of ASP5 during infection and that the conserved RRL motif is critical for cleavage by this protease.

## MATERIALS AND METHODS

### *Toxoplasma* transfection and growth.

Candidate genes were tagged endogenously within parasites using the CRISPR/Cas9 system which has been adapted for use in *Toxoplasma* (49, 50). Briefly, genes were tagged just prior to the endogenous stop codon following guide selection from EuPaGDT (http://grna.ctegd.uga.edu/batch_tagging.html). The CRISPR target plasmid (made by Q5 mutagenesis [NEB]) was cotransfected with homologous repair constructs containing Ty or HA epitope tag as previously described (21). Two oligonucleotides with at least 30 bp of complementarity at their 3′ end, usually over the HA or TY epitope, were annealed together in IDT-duplex buffer by heating to 98°C for 2 min and then gradually allowed to cool (49). Ten micrograms of Cas9 plasmid was combined with the total 80 μg of annealed oligonucleotides and resuspended in 20 μl P3 solution (Lonza) in an Amaxa 4D Nucleofector (Lonza) using the code FI-115 (Human Unstimulated T-cells). Human foreskin fibroblasts (HFF) were grown to confluence in Dulbecco’s modified Eagle medium (DMEM), supplemented with 10% Cosmic Calf serum (HyClone), and refreshed with DMEM with 1% fetal calf serum when inoculated with parasites.

### PCR and plasmid construction.

Please consult supplemental material for details.

### Immunofluorescence assays (IFAs) and microscopy.

Fixation, preparation of reagents, and mounting for IFAs were performed as previously described (21). All images were captured on the Zeiss LSM 880 equipped with Airyscan detector. Before each session, channel alignment was performed using a FocalCheck fluorescence microscope test slide 1 (ThermoFisher), and subsequent images were automatically aligned in FIJI using the plug-in TransformJ Translate.

### Western blot.

Immunoblot samples were pelleted and then lysed for 30 min at 4°C in 1% (vol/vol) Triton X-100, 1 mM MgCl_2_ in PBS (Gibco) supplemented with final 1× cOmplete protease inhibitors (Sigma) and 0.2% (vol/vol) Benzonase (Merck). Samples were then combined with an equal volume of 2× sample buffer, and 20 μl loaded onto a gel. Proteins were transferred onto nitrocellulose and then blocked in 5% (wt/vol) milk in 0.05% Tween 20-supplemented PBS. Primary and secondary antibodies were diluted in milk/PBS solution. For LI-COR Western blots, the membranes were imaged on an Odyssey Fc imager (LI-COR Biosciences) using IRDye 800CW goat anti-rat, IRDye 800CW goat anti-mouse, and IRDye 680RD goat anti-rabbit antibodies. Antibodies used in this study were anti-HA (αHA) 3F10 (Roche), αHA (rabbit; in-house), αTY1 BB2 (51), αGAP45 (52), αGRA1 (53), αMAF1 (14), αIMC1 (54), and αROP2/3/4 (55).

### Quantitative proteomics.

Please consult the Materials and Methods section in Text S1 in the supplemental material for details for SILAC labeling, protein extraction, TAILS and HYTANE purification, pH fractionation, LC MS/MS, and data analysis.

### Virulence experiments.

All mouse experiments were conducted within the regulations of the Walter and Eliza Hall Institute (WEHI) Animal Ethics Committee (AEC). PruΔ*ku80*Δhx (WT), PruΔ*ku80*Δ*hx*Δ*asp5* (Δ*asp5*), PruΔ*ku80*Δ*hx*Δ*wng1* (Δ*wng1*), and PruΔ*ku80*Δ*hx*Δ*wng2* (Δ*wng2*) parasites were grown in HFFs until ∼10% lysed, then scraped, and released from host cells by passage through a 27-gauge needle. Parasites were then counted and resuspended at the indicated dose in 200 μl PBS and then injected intraperitoneally into six 6- to 8-week-old C57BL/6 mice. Mice were weighed daily and culled after exhibiting body weight loss of greater than 15% for three consecutive days if they were not going to recover (as per the WEHI AEC) or when moribund. All surviving mice were sacrificed 30 days after infection, and the resulting cardiac bleed serum was used as a 1/50 dilution primary antibody to check for seroconversion against tachyzoite lysate.

### Data sets.

The mass spectrometry proteomics data have been deposited to the ProteomeXchange (56) Consortium via the PRIDE (57) partner repository with the data set identifier PXD008574.

10.1128/mBio.01796-18.9 TABLE S3 Summary of the proteins that were found to be significantly enriched in the pairwise comparisons of the 3xHA-tagged bait proteins. Data include the log_2_ protein ratios and *P* values (BH corrected) as well as unique number of sequences used in the label-free quantitation. Download Table S3, XLSX file, 0.01 MB. Copyright © 2018 Coffey et al. 2018 Coffey et al. This content is distributed under the terms of the Creative Commons Attribution 4.0 International license.

10.1128/mBio.01796-18.10 TABLE S4 Oligonucleotides used in this study. Download Table S4, XLSX file, 0.01 MB. Copyright © 2018 Coffey et al. 2018 Coffey et al. This content is distributed under the terms of the Creative Commons Attribution 4.0 International license.
