# Supplementary material for: Aspartyl Protease 5 Matures Dense Granule Proteins That Reside at the Host-Parasite Interface in Toxoplasma gondii
Source: mBio. 2018 Oct 30;9(5):e01796-18. doi: 10.1128/mBio.01796-18 (PMC6212819; doi:10.1128/mBio.01796-18)
Supplement: TEXT S1 [file mbo005184124s1.docx]

**Aspartyl Protease 5 matures dense granule proteins that reside at the host-parasite interface in *Toxoplasma gondii***

Michael J Coffey^1,2^, Laura F Dagley^1,2^, Simona Seizova^1,2^, Eugene A Kapp^1,2^, Giuseppe Infusini^1,2^, David S Roos^3^, Justin A Boddey^1,2^, Andrew I Webb^1,2^ and Christopher J Tonkin^1,2*^.

^1^ – The Walter and Eliza Hall Institute of Medical Research, Melbourne, Australia

^2^ – The Department of Medical Biology, The University of Melbourne, Melbourne, Australia

^3^- Department of Biology, University of Pennsylvania, Philadelphia, PA 19104

^*^For Correspondence:

Chris Tonkin

Division of Infection and Immunity

The Walter and Eliza Hall Institute of Medical Research

1G Royal Parade, Parkville 3052, Victoria, Australia

Ph: +613 9345 2926

Fax: +613 9347 0852

Email: [tonkin@wehi.edu.au](mailto:tonkin@wehi.edu.au)

**Supplementary Results**

***Analysis of TAILS data and gene annotation***

Experiments described in this study identified 1911 distinct N-terminal peptides (excluding 353 exact duplicates, from a total of 2264 peptides).  1606 are unique (excluding 305 overlapping peptides), and map to 1139 annotated *Toxoplasma* genes.  Considering only the most N-terminal peptides for each gene (i.e. excluding 467 downstream peptides), 190 tagged N-terminal Met mapping precisely to the N-terminus of annotated proteins (Table S1).  An additional 242 peptides tagged an amino acid mapping to position 2 of annotated proteins, i.e. one amino acid downstream of the annotated translational start site.  Adjacent sequences are consistent with the *Toxoplasma* translational start consensus: c-A-a/c-a/c/g-ATG- (Table S1), and indicate that *Toxoplasma* harbors aminopeptidase activity that cleaves >99% of Met-Ala- & Met-Ser- peptides, but <1% of Met-Glu- & Met-Asp- peptides (data not shown).  This observation is consistent with a previous report on the importance of the penultimate N-terminal amino acid in regulating *Toxoplasma* translational efficiency (1).  In aggregate, these data serve to validate the current annotation for 432 *Toxoplasma* proteins.

An additional 130 peptides map to a Met -- or 1 amino acid downstream of a Met -- but downstream of the annotated translational start site.  The sequence context for the annotated translational start sites of these proteins is poor, but the sequence context is much better for candidate start sites associated with the TAILS peptides, strongly suggesting that the annotation of these proteins should be reviewed (based on analysis of multiple sequence alignments, RNA-seq data, etc).  These genes have been flagged in the ToxoDB database.

260 peptides map to other amino acids distinct from the annotated translational start sites, but immediately downstream of an Arg amino acid, suggesting that these may be experimental artifacts attributable to incomplete removal of cleavage products introduced during sample preparation.  Consistent with this interpretation, the annotated translational start sites for these proteins matches the genome-wide consensus.

Finally, 317 peptides map to other amino acids: not Met, and not downstream of either Met or Arg.  The annotated translational start for these proteins matches the genome-wide consensus.  These are likely to represent internal proteolytic cleavage sites, such as those mediated by signal peptidase, ASP5, etc.  Consistent with this hypothesis, nearly 50% of these proteins are predicted to encode a secretory signal sequence (more than double the genome as a whole).

**Materials and Methods**

***Genetic strategies***

Candidate genes were tagged endogenously within parasites using the CRISPR/Cas9 system which has been adapted for use in *Toxoplasma* (2, 3). Briefly, genes were tagged just prior to the endogenous stop codon following guide selection from EuPaGDT (http://grna.ctegd.uga.edu/batch_tagging.html). The CRISPR target plasmid (made by Q5 mutagenesis, NEB) was co-transfected with homologous repair constructs containing, TY- or HA-epitope tag as previously described (4). It was noted that endogenous tagging efficiency was greatly increased when flanks of at least 30 base pairs were used and therefore 60 bp annealed oligos (IDT) were used to facilitate this process. To achieve this, two oligos with at least 30 bp of complementarity at their 3’ end, usually over the HA- or TY-epitope, were annealed together in IDT-duplex buffer by heating to 98 °C for two minutes then gradually allowed to cool (2). 10 μg of Cas9 plasmid was combined with the total 80 μg of annealed oligos, then precipitated using EtOH/NaAc prior to transfection. Dried DNA was resuspended in 3 μL of elution buffer (EB, Qiagen), followed by 20 μL P3 solution (Lonza), prior to transfection in a 16-well Nucleocuvette Strip in an Amaxa 4D Nucleofector (Lonza) using the code FI-115 (Human Unstimulated T-cells). For genetic knock outs or for targeted mutations (i.e. RRL 🡪 ARL), the epitope tag within the annealed oligos was exchanged for either a stop codon or a codon encoding alanine, which were than annealed and transfected as above. As transfections using these conditions were found to yield >90% of parasites expressing GFP from the pSAG1-Cas9-GFP-sgUPRT vector (data not shown), we did not employ any drug selection for isolating most parasite lines. Incorporation of either TY- or HA-epitope tagged-parasite lines ranged between 20-90% and were obtained by diluting freshly-transfected parasites directly into 96-well plates (Corning) and screening parasites from wells with single plaques by immunofluorescence or immunoblot. Screening for knock-outs was performed in the same manner, however the homologous recombination template encoded stop codons and induced a frameshift mutation. For genes where tagging had an associated fitness cost, or for some reason did not respond to the above method, we co-transfected the CRISPR-cutting plasmid with a HA_3_-DHFR3’UTR-HXGPRT cassette, flanked with 30 bp of homologous DNA to facilitate integration at the cut site.

RH*Δhx* tachyzoites were transfected with a CRISPR guide designed to disrupt the ku80 gene (sg∆ku80_Cas9_GFP), engineered through Q5 mutagenesis (NEB) of the pSAG1-GFP-Cas9-sgUPRT plasmid (3) with primers #1/2 (Table S3) then cloned into 96 well plates. A resulting clone was chosen (RH*Δku80Δhx*, abbreviated to WT) that had a frameshift mutation within the *ku80* coding region and this was used as the parental line for subsequent experiments. This line was subsequently transfected with pU6-Universal:sgASP5-2 (4) supplemented with annealed oligos #3/4 then sequenced with primers #5/6 to create the line RH*Δku80ΔhxΔasp5* (abbreviated to *Δasp5*) which was used for all subsequent experiments. Both parental lines above were transfected with the plasmid sgLCAT_3’tag_Cas9_GFP, made by Q5 mutagenesis of the parental pSAG1-GFP-Cas9-sgUPRT plasmid (used for all subsequent Q5 reactions) using primers #7 (common Q5 reverse primer) and #8. This transfection was supplemented with 10 μg of Primestar (Takara) PCR product (LCAT5’-HA_3_-DHFR3’UTR-HXGPRT-LCAT3’), amplified with primers #9/10 from the pLIC-3HA-HX vector (5), followed by drug selection and cloning, to yield WT LCAT-HA_3_ and *Δasp5* LCAT-HA_3_ (Figure 3B). sgLCAT_ARL_Cas9_GFP was generated by Q5 mutagenesis with primers #7/11, then supplemented with annealed oligos #12/13 and transfected into WT LCAT-HA_3_ parasites to generate WT LCAT_ARL_-3HA parasites (Figure 3B).

GRA46 was tagged at the C-terminus by transfection with sgGRA46_3’tag_Cas9_GFP (generated with oligos #7/14) and supplemented with oligos #15/16 (Ty tag, Figure 4C) or #17/18 (HA tag, Figure 4B), and at the N-terminus with sgGRA46

_5’tag_Cas9_GFP and oligos #20/21 (Figure 4C). GRA46_ARL_-HA parasites were generated through transfection of sgGRA46_ARL_Cas9_GFP (generated with oligos #7/22) supplemented with oligos #23/24. GRA46 was deleted by transfection with sg∆gra46_Cas9_GFP (Q5 mutagenesis with primers #7/25) supplemented with the homologous template GRA46-5’-LOXP-DHFR-mCherry-LOXP-GRA46-3’ (amplified from the pLOXP-DHFR-mCherry vector (6)) using primers #26/27. Transfection was followed by pyrimethamine selection and transfection with pMin-Cre (7) to excise the DHFR/mCherry cassette to generate Pru*Δku80ΔhxΔgra46* (Figure S1).

WNG1-HA lines were generated by transfection with the plasmid WNG1-3’tag_Cas9_GFP (Q5 with primers #7/28) and the extended oligo (ultramer) #29. Subsequent mutagenesis of these parasites from RRL🡪ARL, resulting in WNG1_ARL_-HA, was generated with the plasmid sgWNG1-ARL_Cas9_GFP (Q5 mutagenesis with primers #7/30) and oligos #31/32 (Figure S1). *Δwng1* parasites were isolated from the same transfection, with one clone sequenced containing a frameshift mutation leading to a premature stop codon (Figure 7A and Figure S1).

WNG2-HA lines were generated using the plasmid sgWNG2-3’tag_Cas9_GFP (Q5 mutagenesis with oligos #7/33) and the oligos #34/35, and subsequent lines mutated from RRL🡪ARL with the plasmid sgWNG2-ARL_Cas9_GFP (Q5 mutagenesis with primers #7/36) and oligos #37/38, resulting in WNG2_ARL_–HA parasites. *Δwng2* parasites were generated with the same plasmid (sgWNG2-ARL_Cas9_GFP) and oligos #39/40 (Figure 7B and Figure S1).

GRA44 was HA-tagged using sgGRA44-3’tag_Cas9_GFP (Q5 mutagenesis with oligos #7/41)

and oligos #42/43, then knocked out using sg*∆gra44*_Cas9_GFP (Q5 mutagenesis with oligos #7/44) and the chloramphenicol cassette, amplified from pgCM3 (8) with oligos #45/46. GRA45-HA lines were generated using sgGRA45-3’tag_Cas9_GFP (Q5 mutagenesis with oligos #7/47) and supplemented with oligos #48/49, then GRA45_ARL_-HA parasites generated through transfection with sgGRA45_ARL__Cas9_GFP (Q5 mutagenesis with oligos #7/50) and oligos #51/52.

***Immunoprecipitations (IPs) and FASP***

HFFs were infected (MOI of 5) with GRA46-HA, GRA44-HA and WNG2-HA, at 36 hpi, parasites were scraped then harvested from large vacuoles, pelleted and lysed by gentle probe tip sonication in 10x pellet volumes of PBS supplemented with 1 % Triton-X 100, 1 mM MgCl_2_, 2x protease inhibitors and 0.2 % Benzonase. Lysed samples were left overnight at 4˚C rolling gently to increase liberation of protein from membranes. Supernatant was then collected following a 10-minute spin at 16 000 *x g* at 4 ˚C, then combined with 80 µL (3x washed) αHA 3F10 agarose beads (Roche Affinity Matrix) for 2 hours at RT. This antibody-lysate mixture was transferred to a chromatography spin column (Bio Rad), pre-moistened with 200 µL PBS. Unbound lysate was left to elute by gravity, then beads were washed with 5 mL 1% Triton-X 100 in PBS, followed by 5 mL PBS only, both left to elute by gravity. Any remaining PBS was removed following a zip spin at 16 000 *x g*, then the butt of the tube was stoppered before addition of 200 µL 0.5 % SDS supplemented with 5 mM fresh DTT in Milli-Q water for 2 minutes at 95˚C. Samples were then spun at 16 000 *x g* for 1 minute at RT into a fresh tube to obtain elute fraction, which was then prepared for mass spectrometry via the filter aided sample preparation (FASP) procedure as previously described (9).

***SILAC labelling and protein extraction***

HFF host cells were grown to confluency as previously described (4). For parasite labelling, tachyzoites were grown in DMEM media for SILAC (Thermo Fisher) supplemented to 1% v/v with dialysed foetal calf serum (FCS) (Invitrogen), and 131 mg/L L-Leucine (Sigma), then for light labelling: 183 mg/L L-Lysine (Sigma) and 75 mg/L L-Arginine (Sigma); for medium labelling: 183 mg/L Lys4 and 75 mg/L Arg6 (Sigma); and for heavy labelling: 183 mg/L Lys8 and 75 mg/L Arg10 (Sigma). Parasites were passed three times to maximize labelling over 5 days, then isolated from large (>32 parasite) vacuoles from three T150cm^2^ flasks per condition, washed 3x in cold PBS, counted, then snap frozen at -80 °C. Parasites were thawed and mixed at a 1:1 WT to *∆asp5* ratio, then lysed in SDS lysis buffer prior to proteins precipitation by methanol/chloroform/water (M/C/W) extraction. Briefly, samples were combined with 3 volumes ice-cold water, 4 volumes of methanol (pre-chilled at -20 °C) and 1 volume chloroform (pre-chilled at -20 °C). Following vigorous centrifugation, samples were spun at >5000 xg for 5 minutes and the upper phase removed, then 4 more volumes of chilled methanol were added before mixing and centrifugation. Following removal of the supernatant, the pellet was washed three times in chilled methanol then dried and resuspended in 6M guanidine hydrochloride (GnHCl) (Sigma).

***TAILS sample preparation***

The extracted SILAC-labelled protein in 6M GnHCl was then diluted to 3M and protein concentration tested using a BCA kit (Thermo Fisher). Samples (4.12 mg for WTH/KOL and 4.72 mg for WTL/KOH) were then prepared following the most current version of the TAILS protocol from the Overall Laboratory (10, 11) (http://clip.ubc.ca/resources/protocols-and-sops/). Briefly, Samples were diluted to 3M GnHCl prior to beginning the protocol, then adjusted to a final concentration of 100 mM HEPES, pH 7.5. Whole proteins were then reduced with 5 mM dithiothreitol (DTT; 60 min, 37˚C) and free cysteines then carbamidomethylated with 10 mM iodoacetamide (IAA; 30 min, dark at RT). Following quenching with 15 mM DTT (20 min, RT), primary amines were dimethylated with 40 mM CH_2_O and 20 mM NaBH_3_CN, adjusted to pH 6.8 with formic acid (FA) then left to incubate (37˚C, overnight). To ensure complete blocking, fresh 20 mM CH_2_O and 10 mM NaBH_3_CN were added for 2 hours at 37˚C, prior to quenching with 100 mM Tris, pH 6.8 at 37˚C for 1 hour. Proteins were then precipitated using M/C/W precipitation (see SILAC labelling paragraph above) then resuspended in 20 uL 100 mM NaOH, followed by HPLC-grade water until the precipitate dissolved, then adjusted to 50 mM HEPES pH 7.2. Samples were then digested with a ratio of 1 mg enzyme :100 mg protein with MS-grade trypsin (Trypsin Gold, Promega) and incubated overnight at 37˚C. A small aliquot of each sample was taken here for the preNTAILS validation (Fig 2A), while the remaining peptide mix was incubated with a 2:1 w/w pre-washed HPG-ALD polymer:peptide ratio, followed by the immediate addition of 20 mM NaBH_3_CN (overnight, 37˚C, pH 6.8) to remove tryptic peptides. After quenching with 100 mM Tris-HCl (pH 6.8, 30 min, 37˚C), unbound peptides representing naturally or experimentally blocked N-termini were recovered in the filtrate after ultra-filtration (Amicon 30 kDa MWCO filters). PreNTAILS and TAILS samples were then desalted with c18 stage tips (12), and the resulting elution frozen at -80˚C then lyophilised, prior to running on the mass spectrometer.

A separate batch of *∆asp5* and WT samples were subjected to reciprocal stable isotope dimethyl labelling with heavy formaldehyde (^13^CD_2_O). Protein aliquots (150 µg) were reduced with DTT, alkylated with IAM and quenched as above. However, fresh deuterated formaldehyde (20 mM final) and light cyanoborohydride (10 mM final) were added and reactions were incubated at 37 °C overnight. The remaining steps were followed as per the TAILS protocol described above.

***HYTANE sample preparation***

Parasites were grown in a T175cm^2^ flask until they had fully egressed and then were scraped, collected and pelleted by centrifugation for 5 min at 2000 RPM. The pellets were washed 3 times in PBS to remove serum then lysed in 10 pellet volumes of 6M GnHCl supplemented with 2 % v/v cOmplete protease inhibitor cocktail (Sigma). These were sonicated using a benchtop probe tip machine, pulsed for 10 seconds on, 10 seconds off for 90 seconds at 15 % amplitude. The supernatant was collected by centrifugation at 17 000 *x* g for 30 minutes at 4 °C. The protein concentration was assessed by BCA (Thermo Fisher) and then the HYTANE method followed for the rest of the protocol (13).

Briefly, 200 µg of protein in 6M GnHCl was adjusted with TEAB to 100 mM pH 8.2, then reduced with DTT (10 mM, 1 hour, 56˚C). Free thiol groups were alkylated with IAA (30 mM, 1 hour at RT in the dark), then extinguished with DTT (10 mM, 20 mins, RT). Primary amines were blocked with 100 mM CH_2_O and 60 mM NaBH_3_CN for 2 hours at 37˚C, then quenched with a final concentration of 1M Tris, pH 7.5. Samples were transferred to Amicon 30 kDa MWCO filters, spun at 14 000 *x* g, retaining a small volume, then washed four times with 250 µL of the ‘wash solution’ (6M GnHCl, 1M Tris pH 8, 50 mM AmBic (NH_4_HCO_3_)). Background solution on the column was exchanged to 50 mM AmBic pH 8.1 which was spun through, then the protein digested with Trypsin Gold (Promega) at 1:00 enzyme to protein (75 µL final volume, 50 mM AmBic, overnight, 37˚C). Digested peptides were eluted, acidified (1% trifluoroacetic acid (TFA)), snap frozen, lyophilised, desalted with c18 stage tips (12) then lyophilised. For hydrophobic tagging of neo-N-termini, peptides were sequentially resuspended in 100 µL MS-grade water, 50 µL 50 mM HEPES (pH 9), 20 µL 600 mM NaBH_3_CN, then 500 µL 10 mg/mL hexadecanal (TCI America, resuspended in 1-propanol), then incubated for 8 hours at 50˚C. To ensure complete labelling, an additional 20 µL (per 100 µg starting protein) of 600 mM NaBH_3_CN was added. Samples were snap frozen, lyophilised, redissolved in 2% acetonitrile (ACN) containing 0.1 % TFA, then hexadecylated peptides were precipitated by centrifugation twice at 17 000 *x* g for 15 minutes. The resulting solution containing blocked primary amines was desalted with c18 stage tips (12) then lyophilised prior before being loading onto the mass spectrometer.

***LC-MS/MS Analysis***

*High pH peptide fractionation*

Purified TAILS peptide samples from each reciprocally labelled sample (WT-H + KO-L and WT-L + KO- H) were reconstituted in 50 μl 5% formic acid and 30 μl subjected to high pH reversed phase analysis on an Agilent 1100 Series HPLC system equipped with a variable wavelength detector (280 nm). Fractionation was performed on XBridge™ Shield C_18_ column (10 x 100 mm, 3.5 μm bead size, Waters). Peptides were separated by their hydrophobicity at a high pH at a flow rate of 0.1 ml/min using a gradient of mobile phase A (5 mM ammonium formate, pH 10) and a mobile phase B (100% acetonitrile, ACN), from 3% to 45% over 57 mins. Fractions were collected every minute across the gradient length and concatenated into 12 fractions. Eluted peptides were dried in a SpeedVac centrifuge and reconstituted in MS loading buffer (2% ACN/0.1% FA) prior to MS analysis.

*Mass spectrometry analysis*

Unfractionated (pre- and post-TAILS) SILAC samples were reconstituted in 25 μl and 50 μl 5% FA, respectively, and 3 μl loaded onto the analytical column in technical triplicate. Fractionated TAILS samples (2 μl) were loaded onto the analytical column. Dimethyl TAILS samples were reconstituted in 25 μl 5% FA and 4 μl loaded onto the analytical column in technical triplicate. Peptides were separated by reverse-phase chromatography on a 1.7 μm C18 fused silica column (I.D. 75 μm, O.D. 360 μm x 25 cm length) packed into an emitter tip (IonOpticks, Australia), using a nano-flow HPLC (M-class, Waters). The HPLC was coupled to an Impact II UHR-QqTOF mass spectrometer (Bruker Daltonics, Bremen) using a CaptiveSpray source and nanoBooster at 0.20 Bar using ACN. Peptides were loaded directly onto the column at a constant flow rate of 400 nL/min with buffer A (99.9% Milli-Q water, 0.1% FA) and eluted with a 90 min linear gradient from 2 to 34% buffer B (99.9% ACN, 0.1% FA). Mass spectra were acquired in a data-dependent manner including an automatic switch between MS and MS/MS scans using a 1.5 second duty cycle and 4 Hz MS1 spectra rate followed by MS/MS scans at 8-20 Hz dependent on precursor intensity for the remainder of the cycle. MS spectra were acquired between a mass range of 200–2000 m/z. Peptide fragmentation was performed using collision-induced dissociation (CID). FASP-digested IP samples and HYTANE samples were analysed by nano-LC-MS/MS on a nanoAcquity system (Waters) coupled to a Q-Exactive mass spectrometer as previously described (9).

*Data analysis*

Raw files consisting of high-resolution MS/MS spectra from the Bruker Impact II instrument were processed with MaxQuant (version 1.5.8.30) for feature detection and protein identification using the Andromeda search engine (14, 15). Extracted peak lists were searched against the *Toxoplasma gondii* ME49 (ToxoDB-26.0) and *Homo sapiens* (UniProt, Oct 2016) databases as well as a separate reverse decoy database to empirically assess the false discovery rate (FDR) using Semispecific free N-term ArgC specificity. The minimum required peptide length was set to 7 amino acids. In the main search, precursor mass tolerance was 0.006 Da and fragment mass tolerance was 40 ppm. Each of the searches were conducted separately. Dimethyl samples: variable modifications of oxidation (methionine), N-terminal acetylation (+42.0105 Da), N-terminal lysine dimethylation (+28.031300), heavy N-terminal lysine dimethylation (+34.06890) and a fixed modification of carbamidomethyl (cysteine). The multiplicity type was set to 2 with light dimethyl lysine (DimethLys0, (+28.031300) and heavy dimethyl lysine (DimethLys6, +34.06890). SILAC samples: variable modifications of oxidation (methionine), N-terminal acetylation (+42.0105 Da), N-terminal lysine dimethylation (+28.031 Da) and a fixed modification of carbamidomethyl (cysteine). The multiplicity type was set to 2 with light lysine dimethylation (DimethLys0,+28.031300) and heavy Arg6 and DimethLys4 (+32.056407). PSM and protein identifications were filtered using a target-decoy approach at an FDR of 1%. HYTANE samples: variable modifications of oxidation (methionine), N-terminal acetylation (+42.0105 Da), N-terminal lysine dimethylation (+28.031300) and a fixed modification of carbamidomethyl (cysteine).

Raw files consisting of high-resolution MS/MS spectra from the Q-Exactive instrument were processed with MaxQuant (version 1.5.6.5) with the settings described above, with the following differences: The search included a strict trypsin specificity allowing up to 2 missed cleavages. Modifications: Carbamidomethylation of Cys was set as a fixed modification, while N-acetylation of proteins, oxidation of Met, the addition of pyroglutamate (at N termini Glu and Gln) were set as variable modifications. The mass tolerance for precursor ions and fragment ions were 20 ppm. HYTANE samples were subjected to further downstream data analysis using in-house software. Specifically, MaxQuant output files were processed and MS2 peak lists converted to Mascot generic format (MGF) using the APLToMGFConverter (https://www.wehi.edu.au/people/andrew-webb/1298/apl-mgf-converter). The MGF files were then analysed using an in-house proteomics search engine (Digger – https://repository.unimelb.edu.au/10187/18167) which forms part of the MSCypher workflow (manuscript in preparation)). Digger was customized in order to output up to 10-pre-and 10-post amino acid residues for all identified peptides. All identified peptides that contained a pre RRL motif were further processed using the Mascot search engine for manual verification and validation. All search parameters and sequence databases were in accordance with those used in the pre-MaxQuant analysis.

***Label-free quantitative proteomics pipeline (IPs)***

Statistically-relevant protein expression changes between the GRA46

vs GRA44 vs WNG2 HA-IPs were identified using a custom in-house designed pipeline as previously described (9) where quantitation was performed at the peptide level. Probability values were corrected for multiple testing using Benjamini–Hochberg method. Cut-off lines with the function y= -log_10_(0.05)+c/(x-x_0_) (16) were introduced to identify significantly enriched proteins. c was set to 0.2 while x_0_ was set to 1, representing proteins with a twofold (log2 protein ratios of 1 or more) or fourfold (log2 protein ratio of 2) change in protein expression, respectively. Supplementary Table 2 contains a summary of the proteins identified with significant differential expression changes.

**References:**

1. Matrajt M, Nishi M, Fraunholz MJ, Peter O, Roos DS. 2002. Amino-terminal control of transgenic protein expression levels in *Toxoplasma gondii*. Mol Biochem Parasitol 120:285-9.

2. Sidik SM, Hackett CG, Tran F, Westwood NJ, Lourido S. 2014. Efficient genome engineering of *Toxoplasma gondii* using CRISPR/Cas9. PLoS One 9:e100450.

3. Shen B, Brown KM, Lee TD, Sibley LD. 2014. Efficient gene disruption in diverse strains of *Toxoplasma gondii* using CRISPR/CAS9. MBio 5:e01114-14.

4. Coffey MJ, Sleebs BE, Uboldi AD, Garnham A, Franco M, Marino ND, Panas MW, Ferguson DJ, Enciso M, O'Neill MT, Lopaticki S, Stewart RJ, Dewson G, Smyth GK, Smith BJ, Masters SL, Boothroyd JC, Boddey JA, Tonkin CJ. 2015. An aspartyl protease defines a novel pathway for export of *Toxoplasma* proteins into the host cell. Elife 4.

5. Gould SB, Kraft LG, van Dooren GG, Goodman CD, Ford KL, Cassin AM, Bacic A, McFadden GI, Waller RF. 2011. Ciliate pellicular proteome identifies novel protein families with characteristic repeat motifs that are common to alveolates. Mol Biol Evol 28:1319-31.

6. Long S, Wang Q, Sibley LD. 2016. Analysis of Noncanonical Calcium-Dependent Protein Kinases in *Toxoplasma gondii* by Targeted Gene Deletion Using CRISPR/Cas9. Infect Immun 84:1262-73.

7. Heaslip AT, Nishi M, Stein B, Hu K. 2011. The motility of a human parasite, *Toxoplasma gondii*, is regulated by a novel lysine methyltransferase. PLoS Pathog 7:e1002201.

8. Katris NJ, van Dooren GG, McMillan PJ, Hanssen E, Tilley L, Waller RF. 2014. The apical complex provides a regulated gateway for secretion of invasion factors in *Toxoplasma*. PLoS Pathog 10:e1004074.

9. Delconte RB, Kolesnik TB, Dagley LF, Rautela J, Shi W, Putz EM, Stannard K, Zhang JG, Teh C, Firth M, Ushiki T, Andoniou CE, Degli-Esposti MA, Sharp PP, Sanvitale CE, Infusini G, Liau NP, Linossi EM, Burns CJ, Carotta S, Gray DH, Seillet C, Hutchinson DS, Belz GT, Webb AI, Alexander WS, Li SS, Bullock AN, Babon JJ, Smyth MJ, Nicholson SE, Huntington ND. 2016. CIS is a potent checkpoint in NK cell-mediated tumor immunity. Nat Immunol 17:816-24.

10. Kleifeld O, Doucet A, auf dem Keller U, Prudova A, Schilling O, Kainthan RK, Starr AE, Foster LJ, Kizhakkedathu JN, Overall CM. 2010. Isotopic labeling of terminal amines in complex samples identifies protein N-termini and protease cleavage products. Nat Biotechnol 28:281-8.

11. Doucet A, Kleifeld O, Kizhakkedathu JN, Overall CM. 2011. Identification of proteolytic products and natural protein N-termini by Terminal Amine Isotopic Labeling of Substrates (TAILS). Methods Mol Biol 753:273-87.

12. Rappsilber J, Mann M, Ishihama Y. 2007. Protocol for micro-purification, enrichment, pre-fractionation and storage of peptides for proteomics using StageTips. Nat Protoc 2:1896-906.

13. Chen L, Shan Y, Weng Y, Sui Z, Zhang X, Liang Z, Zhang L, Zhang Y. 2016. Hydrophobic Tagging-Assisted N-Termini Enrichment for In-Depth N-Terminome Analysis. Anal Chem 88:8390-5.

14. Cox J, Neuhauser N, Michalski A, Scheltema RA, Olsen JV, Mann M. 2011. Andromeda: a peptide search engine integrated into the MaxQuant environment. J Proteome Res 10:1794-805.

15. Cox J, Mann M. 2008. MaxQuant enables high peptide identification rates, individualized p.p.b.-range mass accuracies and proteome-wide protein quantification. Nat Biotechnol 26:1367-72.

16. Keilhauer EC, Hein MY, Mann M. 2015. Accurate protein complex retrieval by affinity enrichment mass spectrometry (AE-MS) rather than affinity purification mass spectrometry (AP-MS). Mol Cell Proteomics 14:120-35.
