# Supplementary material for: Aspartyl Protease 5 Matures Dense Granule Proteins That Reside at the Host-Parasite Interface in Toxoplasma gondii
Source: mBio. 2018 Oct 30;9(5):e01796-18. doi: 10.1128/mBio.01796-18 (PMC6212819; doi:10.1128/mBio.01796-18)
Supplement: TABLE S1 [file mbo005184124st1.pdf]

AA-1	TAILS Peptide AA1	pos'n sition in CDS	# of Genes	Annotated ATG context xAxx-ATG		cA(ac)(acg)-ATG		Signal Peptide Annotation		Peptide AA1 context (if different) xAxx-AA1		cA(ac)(acg)-AA1		Notes
≠M≠R	**M**	**1**	**175**	112	***64%***	60	***34%***	10	***6%***					***N-terminal tagging supports existing annotation;*** *not cleaved by aminopeptidase (89/175 Met-Glu/Asp)*
R	**M**	**1**	**15**	14	***93%***			1	***7%***					***N-terminal tagging supports existing annotation;*** *not cleaved by aminopeptidase (8/15 Met-Glu/Asp); or could be Arg cleavage product from sample prep, but upstream context is usually AGr (A@-3) rather than*
M	***any***	**2**	**242**	180	***74%***	85	***35%***	20	***8%***	242	***100%***			***N-terminal tagging supports existing annotation;*** *cleaved by aminopeptidase (197/242 Met-Ala/Ser)*
≠M≠R	**M**	**>2**	**48**	12	***25%***	0	***0%***	8	***17%***	31	***65%***	17	***35%***	***N-terminal tagging suggests probable alternative gene models;*** *mapped peptide ATG context is more likely to be translated than annotated ATG context; not cleaved by aminopeptidase (19/48 Met-Glu/Asp)*
R	**M**	**>2**	**17**	11	***65%***	6	***35%***	6	***35%***	6	***35%***			***N-terminal tagging suggests possible alternative gene models,*** *although annotated ATG context is more likely to be translated than mapped peptide ATG context; not cleaved by aminopeptidase (4/17 Met-Glu/*
M	***any***	**>2**	**65**	19	***29%***	5	***8%***	15	***23%***	65	***100%***			***N-terminal tagging suggests probable alternative gene models;*** *mapped peptide ATG context is more likely to be translated than annotated ATG context; cleaved by aminopeptidase (57/65 Met-Ala/Ser)*
≠M≠R	**≠M**	**>2**	**317**	188	***59%***	87	***27%***	145	***46%***	46	***15%***	8	***3%***	***N-terminal tagging identifies probable internal proteolytic cleavage sites;*** *supported by ATG context & high prevalence of signal peptide predictions*
R	**≠M**	**>2**	**260**	169	***65%***	89	***34%***	68	***26%***	54	***21%***			***Likely experimental artefacts,*** *attributable to incomplete removal of experimental proteolytic cleavage products*
any	**M**	**1**	**8244**	4433	***54%***	1702	***21%***	1939	***24%***	***Entire Annotated Genome***				

**2264**	***N-terminally-tagged peptides (from all experiments)***
**1911**	***Distinct peptides (excluding 353 exact duplicates)***
**1606**	***Unique peptides (excluding 305 overlapping peptides)***
**1139**	***Number of genes (excluding 467 downstream peptides)***
