# Supplementary figures and images for: Aspartyl Protease 5 Matures Dense Granule Proteins That Reside at the Host-Parasite Interface in Toxoplasma gondii
Source: mBio. 2018 Oct 30;9(5):e01796-18. doi: 10.1128/mBio.01796-18 (PMC6212819; doi:10.1128/mBio.01796-18)

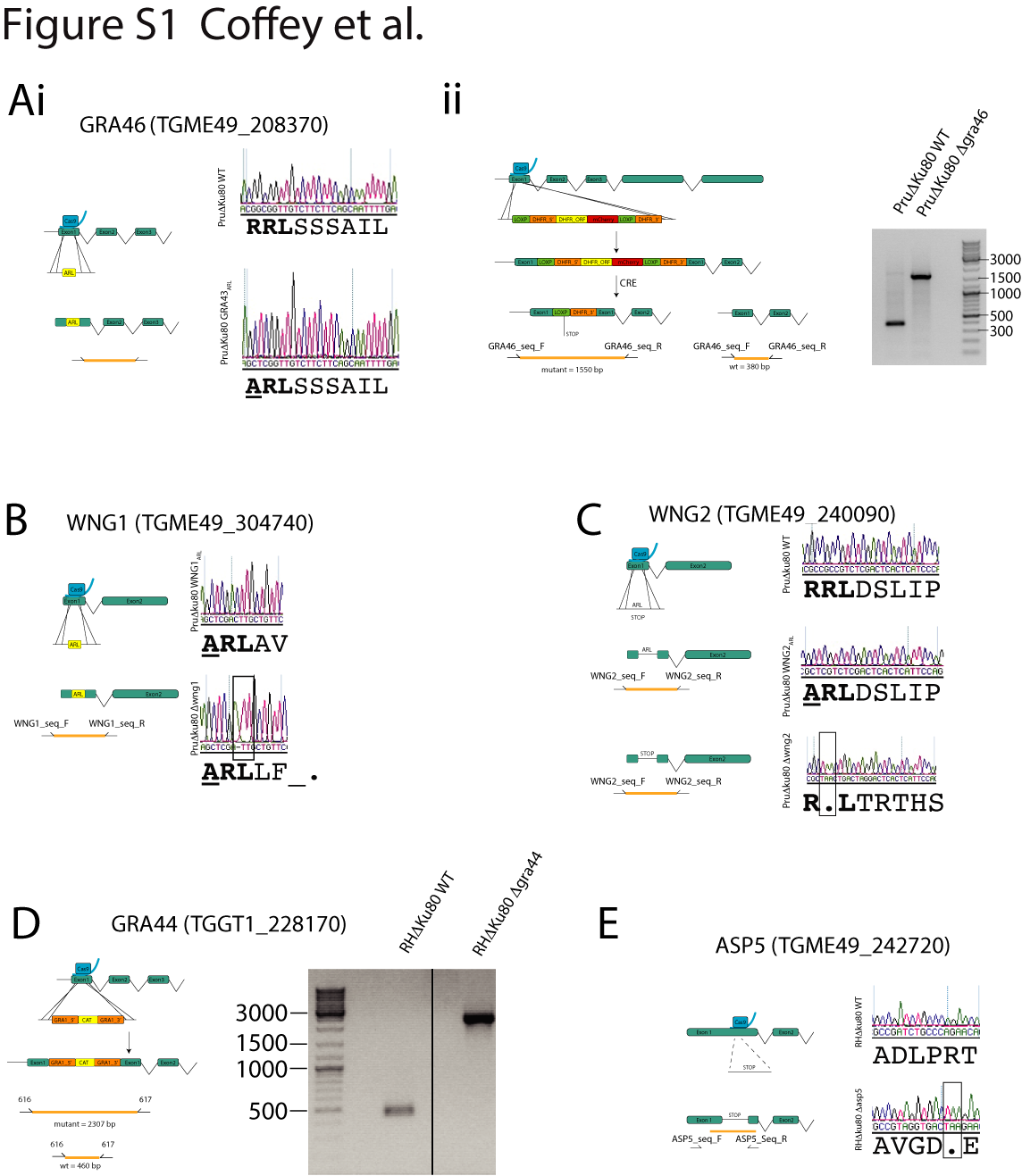

Supplement: FIG S1 [file mbo005184124sf1.tif]

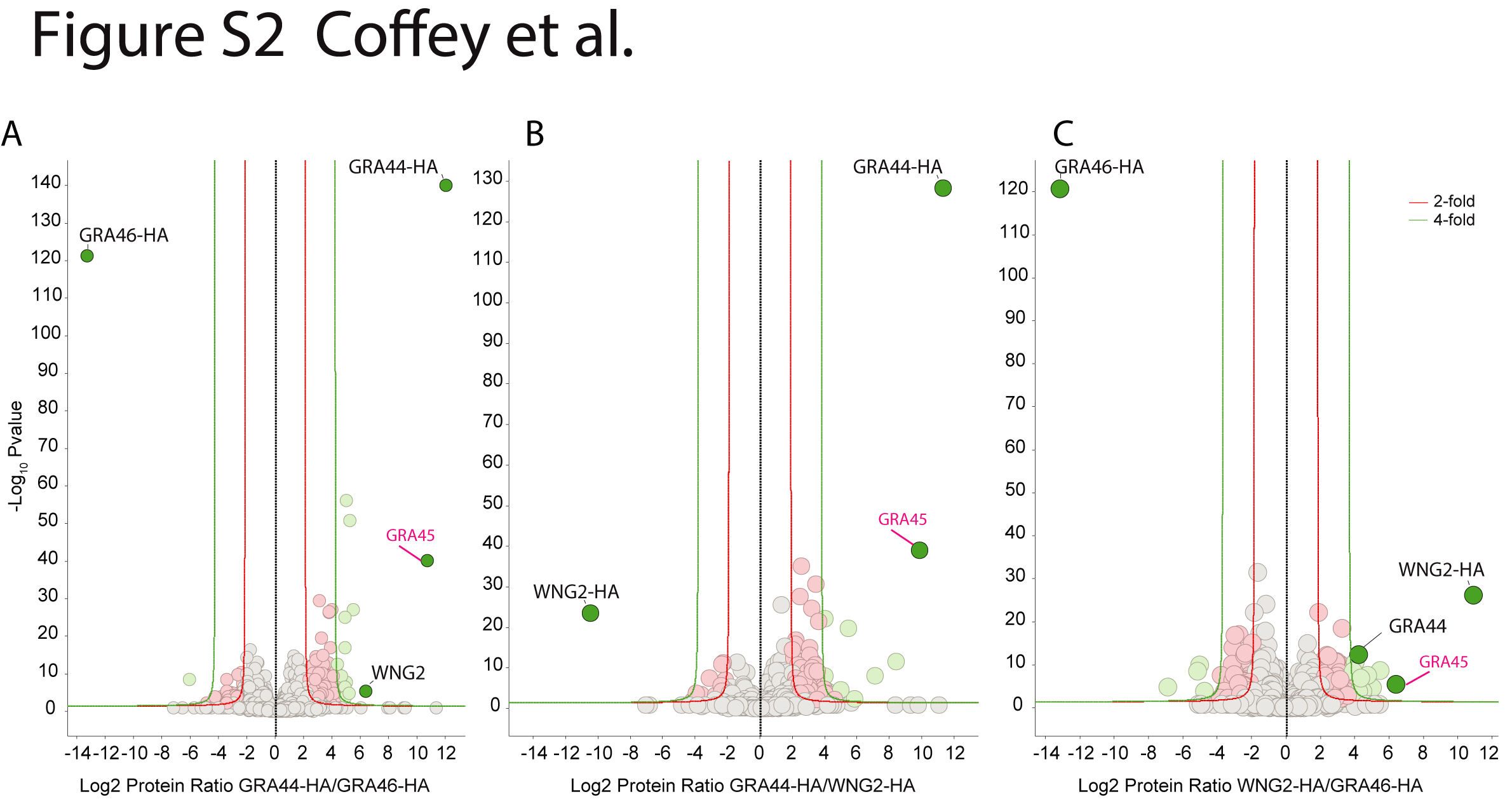

Supplement: FIG S2 [file mbo005184124sf2.tif]

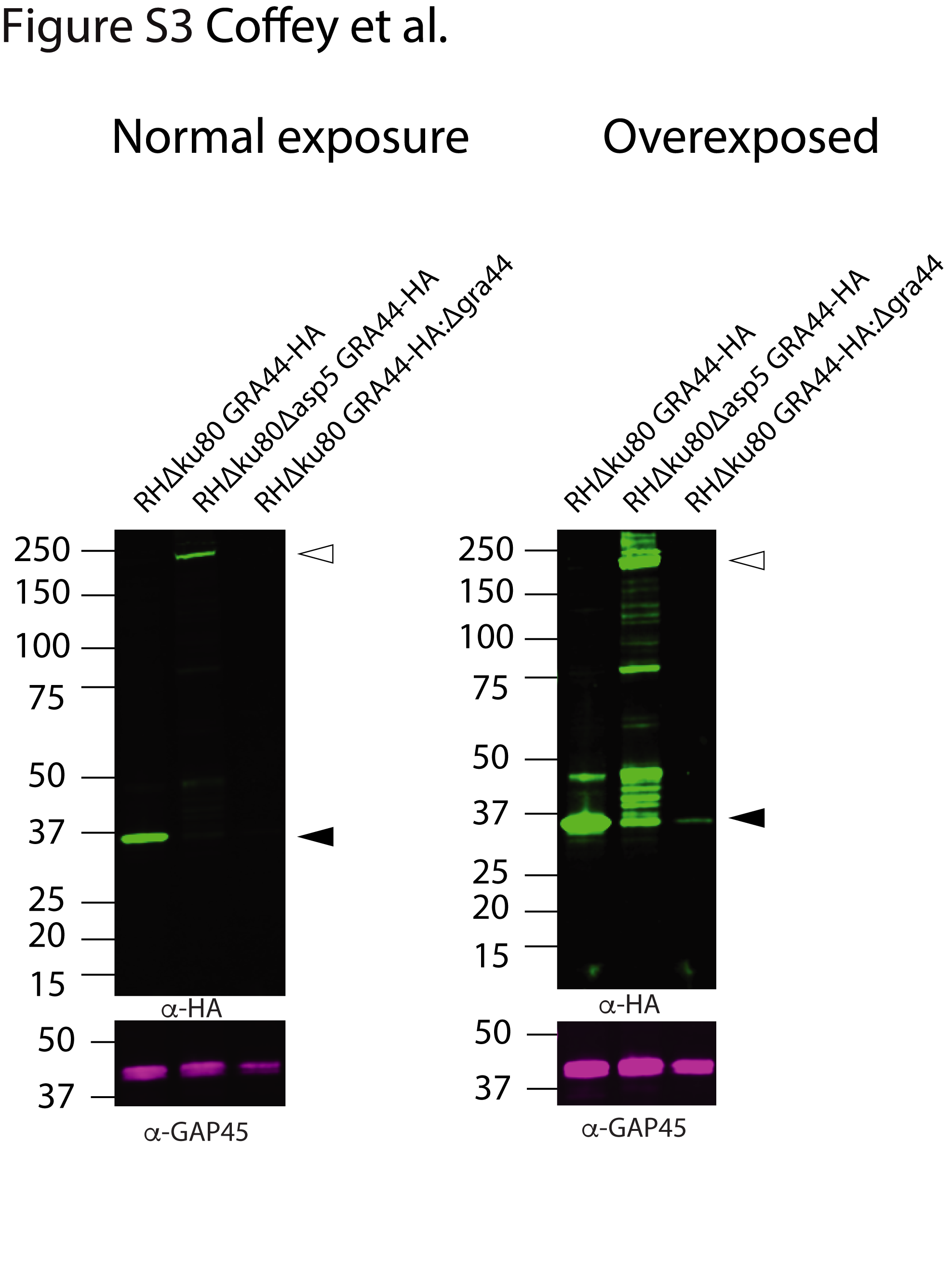

Supplement: FIG S3 [file mbo005184124sf3.tif]

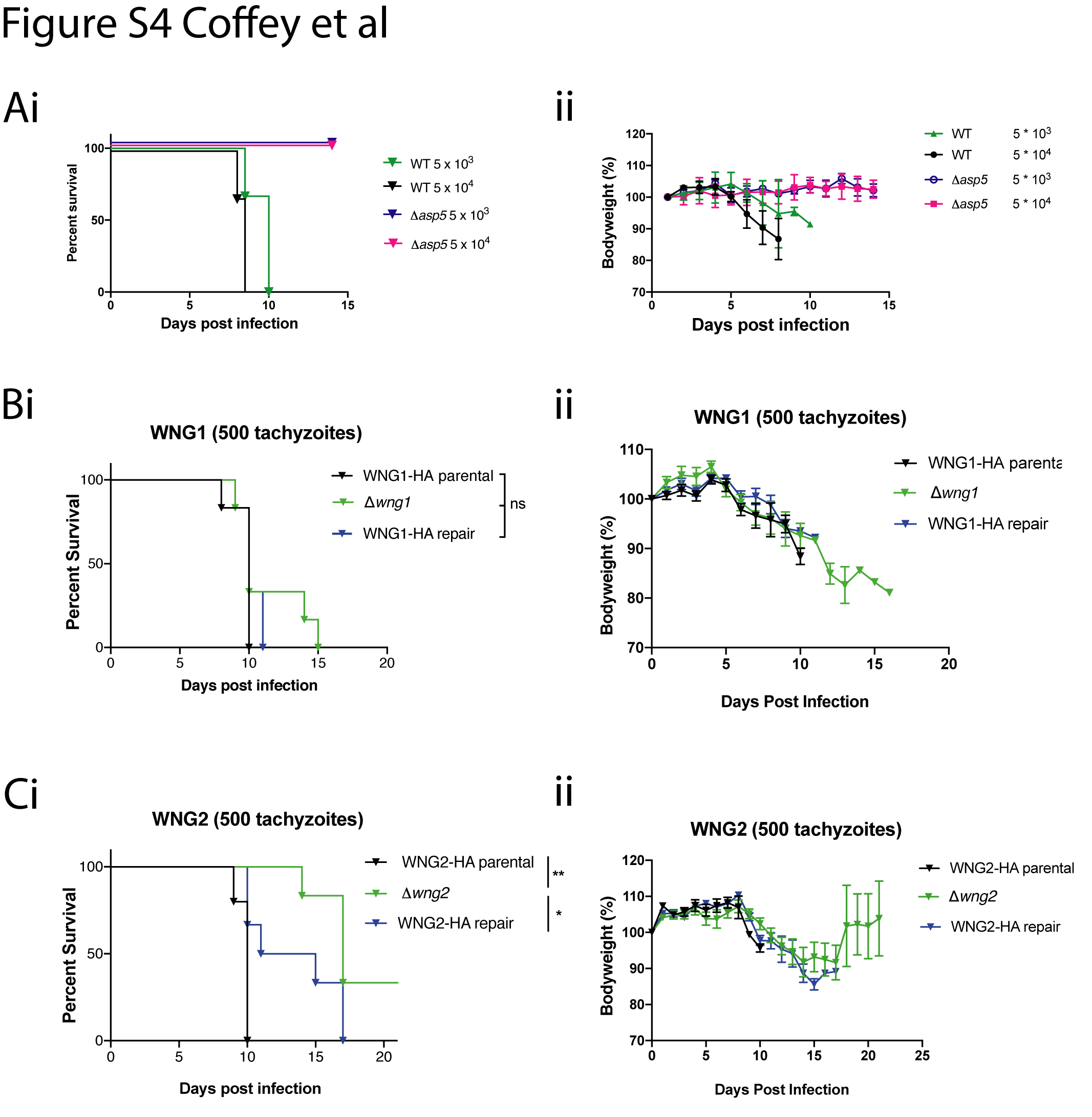

Supplement: FIG S4 [file mbo005184124sf4.tif]
