# Supplementary material for: Aspartyl Protease 5 Matures Dense Granule Proteins That Reside at the Host-Parasite Interface in Toxoplasma gondii
Source: mBio. 2018 Oct 30;9(5):e01796-18. doi: 10.1128/mBio.01796-18 (PMC6212819; doi:10.1128/mBio.01796-18)
Supplement: FIG S5 [file mbo005184124sf5.pdf]

Coffey *et al.* Figure S5-LFQ (HYTANE) Spectra

Peptide View

MS/MS Fragmentation of **SSSAILTGQQIGTYR**
Found in **TGME49_208370** in **TgondiiME49**, TGME49_208370 | organism=Toxoplasma_gondii_ME49 | product=myosin heavy chain, putative | location=TGME49_chrIb:636374-641145(+) | length=1124 | sequence_SO=chromosome | SO=protein_coding

Match to Query 35055: 1608.845450 from(805.430001,2+) intensity(77035000.0000) rtinseconds(5020.8) scans(17400) index(13152)
Title: RawFile: P3308_WT_1_3_1 Index: 17400 Precursor: 0 _multi_ Charge: 2 FeatureIntensity: 77035000 Feature#: 857456 RtApex: 5026.38 FeaturePif: 0.9376948 MS2Pif: 0.9142784 Ndp: 116 Ns: 34 Nip: 6 Seq: SSSAILTGQQIGTYR Score: 225.97 #MS2: 1
Local Instrument: Q-Exactive_Internal
Data file C:\temp\mgf\Coffey\P3308_WT_1_3_1.mgf

Click mouse within plot area to zoom in by factor of two about that point
Or, Plot from 100 to 1700 Da Full range
Label all possible matches Label matches used for scoring
Show Y-axis

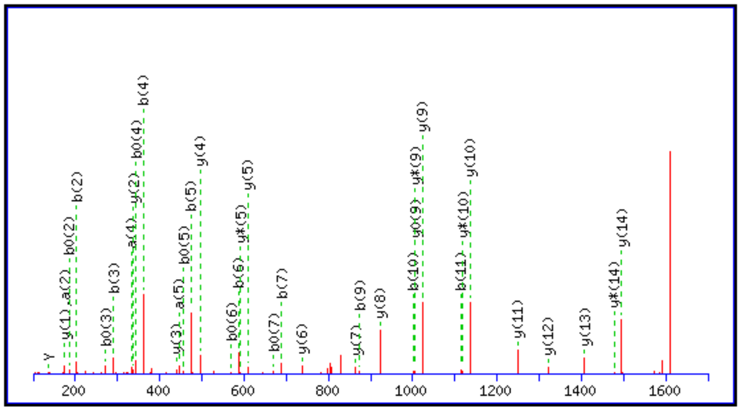

Monoisotopic mass of neutral peptide Mr(calc): 1608.8471
Fixed modifications: Carbamidomethyl (C) (apply to specified residues or termini only)
Variable modifications:
N-term : Dimethyl (N-term)
Ions Score: 123 **Expect:** 2.4e-10
Matches : 38/231 fragment ions using 57 most intense peaks (help)

Peptide View

MS/MS Fragmentation of **DSLIPGFLKR**
Found in **TGME49_240090** in **TgondiiME49**, TGME49_240090 | organism=Toxoplasma_gondii_ME49 | product=rhoptry kinase family protein ROP34, putative | location=TGME49_chrVI:1057698-1060095(+) | length=553 | sequence_SO=chromosome | SO=protein_coding

Match to Query 19373: 1200.722037 from(401.247955,3+) intensity(30253000.0000) rtinseconds(5915.58) scans(26590) index(6467)
Title: RawFile: P3308_WT_1_2_1 Index: 26590 Precursor: 0 _multi_ Charge: 3 FeatureIntensity: 30253000 Feature#: 622473 RtApex: 5918.88 FeaturePif: 0.9254783 MS2Pif: 0.8905471 Ndp: 45 Ns: 18 Nip: 3 Seq: NaN Score: NaN #MS2: 1
Local Instrument: Q-Exactive_Internal
Data file C:\temp\mgf\Coffey\P3308_WT_1_2_1.mgf

Click mouse within plot area to zoom in by factor of two about that point
Or, Plot from 100 to 1600 Da Full range
Label all possible matches Label matches used for scoring
Show Y-axis

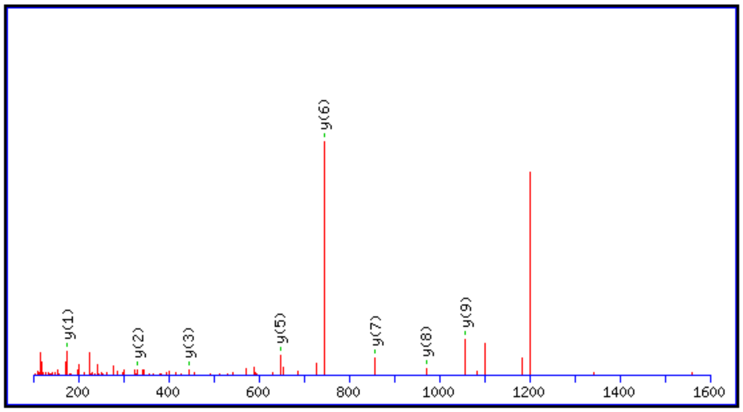

Monoisotopic mass of neutral peptide Mr(calc): 1200.7230
Fixed modifications: Carbamidomethyl (C) (apply to specified residues or termini only)
Variable modifications:
N-term : Dimethyl (N-term)
K9 : Dimethyl (K)
Ions Score: 75 **Expect:** 1.7e-06

Peptide View

MS/MS Fragmentation of **SGIIKTLVLWDPVQR**
Found in **TGME49_228170** in **TgondiiME49**, TGME49_228170 | organism=Toxoplasma_gondii_ME49 | product=inner membrane complex protein IMC2A (IMC2A) | location=TGME49_chrX:520228-525722(-) | length=1491 | sequence_SO=chromosome | SO=protein_coding

Match to Query 27316: 1794.043068 from(599.021632,3+) intensity(86180000.0000) rtinseconds(7272) scans(30456) index(11954)
Title: RawFile: P3308_WT_1_7_1 Index: 30456 Precursor: 0 _multi_ Charge: 3 FeatureIntensity: 86180000 Feature#: 1331976 RtApex: 7274.04 FeaturePif: 0.9936985 MS2Pif: 0.9952576 Ndp: 63 Ns: 14 Nip: 6 Seq: NaN Score: NaN #MS2: 1
Local Instrument: Q-Exactive_Internal
Data file C:\temp\mgf\Coffey\P3308_WT_1_7_1.mgf

Click mouse within plot area to zoom in by factor of two about that point
Or, Plot from 100 to 1500 Da Full range
Label all possible matches Label matches used for scoring
Show Y-axis

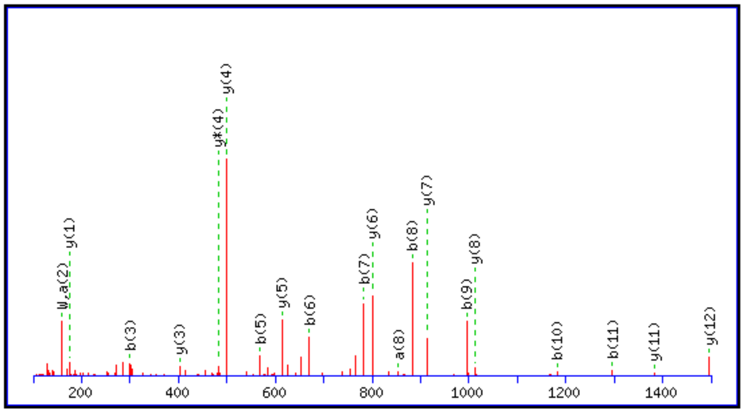


```
Monoisotopic mass of neutral peptide Mr(calc): 1794.0403
Fixed modifications: Carbamidomethyl (C) (apply to specified residues or termini only)
Variable modifications:
N-term : Acetyl (N-term)
K5     : Dimethyl (K)
Ions Score: 83  Expect: 2e-07
Matches : 21/243 fragment ions using 25 most intense peaks   (help)
```

Peptide View

MS/MS Fragmentation of **MFAAAPLQSFSVTNKQFHPEGLEAQAPRPHQGLDMR**
Found in **TGME49_286530** in **TgondiiME49**, TGME49_286530 | organism=Toxoplasma_gondii_ME49 | product=hypothetical protein | location=TGME49_chrV:1791334-1792982(+) | length=218 | sequence_SO=chromosome | SO=protein_coding

Match to Query 48814: 4062.033360 from(678.012836,6+) intensity(177330000.0000) rtinseconds(6772.8) scans(29578) index(22606)
Title: RawFile: P3308_ASP5_1_1_1 Index: 29578 Precursor: 0 _multi_ Charge: 6 FeatureIntensity: 177330000 Feature#: 1722982 RtApex: 6757.14 FeaturePif: 0.8087286 MS2Pif: 0.62454 Ndp: 297 Ns: 44 Nip: 9 Seq: NaN Score: NaN #MS2: 1
Local Instrument: Q-Exactive_Internal
Data file C:\temp\mgf\Coffey\P3308_ASP5_1_1_1.mgf

Click mouse within plot area to zoom in by factor of two about that point
Or, [Plot from] [0] to [3800] Da [Full range]
Label all possible matches ○ Label matches used for scoring ◉
Show Y-axis ☐

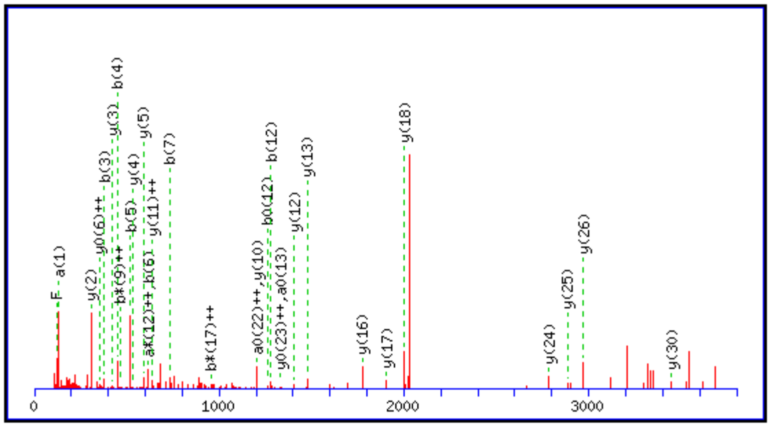


```
Monoisotopic mass of neutral peptide Mr(calc): 4062.0356
Fixed modifications: Carbamidomethyl (C) (apply to specified residues or termini only)
Variable modifications:
N-term : Dimethyl (N-term)
K15    : Dimethyl (K)
Ions Score: 47  Expect: 4.1e-05
Matches : 33/602 fragment ions using 71 most intense peaks   (help)
```

Peptide View

MS/MS Fragmentation of **QAGVYFSEEDR**
Found in **TGME49_233695** in **TgondiiME49**, TGME49_233695 | organism=Toxoplasma_gondii_ME49 | product=hypothetical protein | location=TGME49_chrVIII:2729119-2731334(-) | length=175 | sequence_SO=chromosome | SO=protein_coding

Match to Query 21064: 1282.546734 from(642.280643,2+) intensity(4441200.0000) rtinseconds(4854.84) scans(16818) index(8061)
Title: RawFile: P3308_WT_1_6_1 Index: 16818 Precursor: 0 _multi_ Charge: 2 FeatureIntensity: 4441200 Feature#: 1219593 RtApex: 4857.9 FeaturePif: 0.9925126 MS2Pif: 0.9126611 Ndp: 58 Ns: 25 Nip: 3 Seq: NaN Score: NaN #MS2: 1
Local Instrument: Q-Exactive_Internal
Data file C:\temp\mgf\Coffey\P3308_WT_1_6_1.mgf

Click mouse within plot area to zoom in by factor of two about that point
Or, Plot from 100 to 1150 Da Full range
Label all possible matches Label matches used for scoring
Show Y-axis

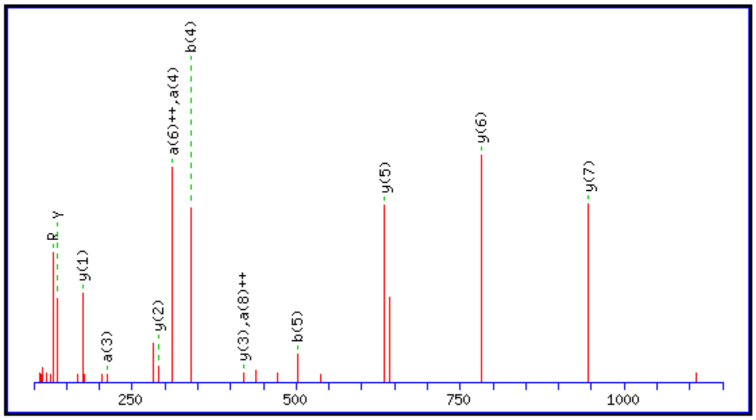


```
Monoisotopic mass of neutral peptide Mr(calc): 1282.5466
Fixed modifications: Carbamidomethyl (C) (apply to specified residues or termini only)
Variable modifications:
N-term : Gln->pyro-Glu (N-term Q)
Ions Score: 44  Expect: 0.00088
Matches : 14/165 fragment ions using 19 most intense peaks   (help)
```

Peptide View

MS/MS Fragmentation of **TTLASTLSLSR**
Found in **TGME49_297890** in **TgondiiME49**, TGME49_297890 | organism=Toxoplasma_gondii_ME49 | product=hypothetical protein | location=TGME49_chrII:2096164-2100807(+) | length=1547 | sequence_SO=chromosome | SO=protein_coding

Match to Query 18739: 1176.671686 from(589.343119,2+) intensity(20307000.0000) rtinseconds(5488.02) scans(20513) index(6570)
Title: RawFile: P3308_WT_1_3_1 Index: 20513 Precursor: 0 _multi_ Charge: 2 FeatureIntensity: 20307000 Feature#: 825566 RtApex: 5490.42 FeaturePif: 0.9101502 MS2Pif: 0.8832461 Ndp: 64 Ns: 24 Nip: 4 Seq: TTLASTLSLSR Score: 107.79 #MS2: 1
Local Instrument: Q-Exactive_Internal
Data file C:\temp\mgf\Coffey\P3308_WT_1_3_1.mgf

Click mouse within plot area to zoom in by factor of two about that point
Or, Plot from 100 to 1200 Da Full range
Label all possible matches Label matches used for scoring
Show Y-axis

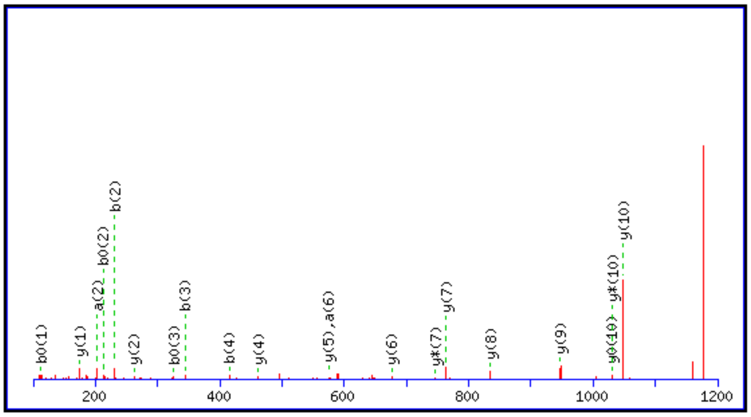

Monoisotopic mass of neutral peptide Mr(calc): 1176.6714
Fixed modifications: Carbamidomethyl (C) (apply to specified residues or termini only)
Variable modifications:
N-term : Dimethyl (N-term)
Ions Score: 65 **Expect:** 4.8e-05
Matches : 20/149 fragment ions using 37 most intense peaks (help)

Peptide View

MS/MS Fragmentation of **YAPGASVVESPVFGTPPSR**
Found in **TGME49_248450** in **TgondiiME49**, TGME49_248450 | organism=Toxoplasma_gondii_ME49 | product=zinc finger, C3HC4 type (RING finger) domain-containing protein | location=TGME49_chrXII:3861774-3868161(+) | length=794 | sequence_SO=chromosome | SO=protein_coding

Match to Query 30830: 1958.971988 from(980.493270,2+) intensity(7636900.0000) rtinseconds(6291) scans(23104) index(13851)
Title: RawFile: P3308_WT_1_1_1 Index: 23104 Precursor: 0 _multi_ Charge: 2 FeatureIntensity: 7636900 Feature#: 580868 RtApex: 6290.58 FeaturePif: 0.6866665 MS2Pif: 0.6866665 Ndp: 43 Ns: 15 Nip: 5 Seq: NaN Score: NaN #MS2: 1
Local Instrument: Q-Exactive_Internal
Data file C:\temp\mgf\Coffey\P3308_WT_1_1_1.mgf

Click mouse within plot area to zoom in by factor of two about that point
Or, Plot from 0 to 2400 Da Full range
Label all possible matches Label matches used for scoring
Show Y-axis

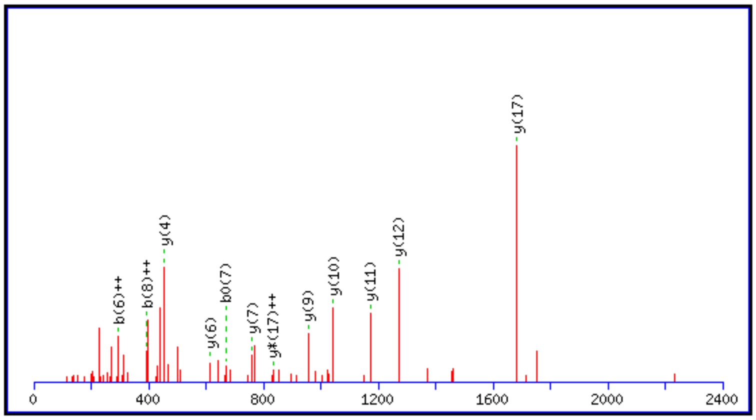

Monoisotopic mass of neutral peptide Mr(calc): 1958.9738
Fixed modifications: Carbamidomethyl (C) (apply to specified residues or termini only)
Variable modifications:
N-term : Acetyl (N-term)
Ions Score: 36 **Expect:** 0.00064
Matches : 12/249 fragment ions using 28 most intense peaks (help)

Peptide View

MS/MS Fragmentation of **AHAEHPEDSATNFLFSFAENSLANREPPEDSAARPSSR**
Found in **TGME49_240090** in **TgondiiME49**, TGME49_240090 | organism=Toxoplasma_gondii_ME49 | product=rhoptry kinase family protein ROP34, putative | location=TGME49_chrVI:1057698-1060095(+) | length=553 | sequence_SO=chromosome | SO=protein_coding

Match to Query 47031: 4195.927490 from(840.192774,5+) intensity(49787000.0000) rtinseconds(6996) scans(29677) index(21300)
Title: RawFile: P3308_ASP5_1_7_1 Index: 29677 Precursor: 0 _multi_ Charge: 5 FeatureIntensity: 49787000 Feature#: 244033 RtApex: 6995.7 FeaturePif: 0.9438615 MS2Pif: 0.9488879 Ndp: 81 Ns: 19 Nip: 8 Seq: NaN Score: NaN #MS2: 1
Local Instrument: Q-Exactive_Internal
Data file C:\temp\mgf\Coffey\P3308_ASP5_1_7_1.mgf

Click mouse within plot area to zoom in by factor of two about that point
Or, Plot from 0 to 4000 Da Full range
Label all possible matches Label matches used for scoring
Show Y-axis

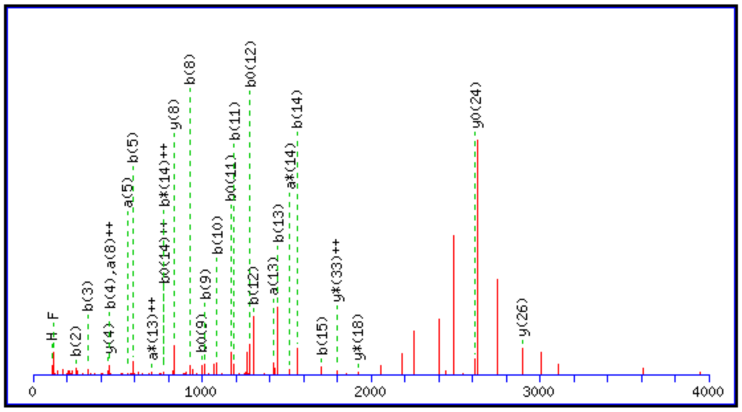


```
Monoisotopic mass of neutral peptide Mr(calc): 4195.9219
Fixed modifications: Carbamidomethyl (C) (apply to specified residues or termini only)
Variable modifications:
N-term : Acetyl (N-term)
Ions Score: 57  Expect: 9.3e-06
Matches : 33/646 fragment ions using 48 most intense peaks   (help)
```

Peptide View

MS/MS Fragmentation of **AHAEHPEDSATNFLFSFAENSLANR**
Found in **TGME49_240090** in **TgondiiME49**, TGME49_240090 | organism=Toxoplasma_gondii_ME49 | product=rhoptry kinase family protein ROP34, putative | location=TGME49_chrVI:1057698-1060095(+) | length=553 | sequence_SO=chromosome | SO=protein_coding

Match to Query 58362: 2816.285793 from(939.769207,3+) intensity(27279000.0000) rtinseconds(7099.8) scans(34762) index(24327)
Title: RawFile: P3308_ASP5_1_4_1 Index: 34762 Precursor: 0 _multi_ Charge: 3 FeatureIntensity: 27279000 Feature#: 2135089 RtApex: 7099.62 FeaturePif: 0.9796388 MS2Pif: 0.9779148 Ndp: 39 Ns: 10 Nip: 5 Seq: NaN Score: NaN #MS2: 1
Local Instrument: Q-Exactive_Internal
Data file C:\temp\mgf\Coffey\P3308_ASP5_1_4_1.mgf

Click mouse within plot area to zoom in by factor of two about that point
Or, Plot from 0 to 3000 Da Full range
Label all possible matches ○ Label matches used for scoring ◉
Show Y-axis ☐

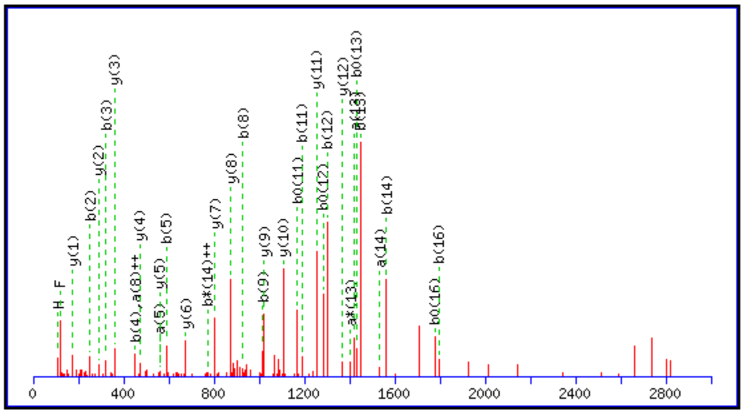


```
Monoisotopic mass of neutral peptide Mr(calc): 2816.2790
Fixed modifications: Carbamidomethyl (C) (apply to specified residues or termini only)
Variable modifications:
N-term : Acetyl (N-term)
Ions Score: 101  Expect: 3.7e-10
Matches : 38/393 fragment ions using 57 most intense peaks   (help)
```
